# Ancient variation of the *AvrPm17* gene in powdery mildew limits the effectiveness of the introgressed rye *Pm17* resistance gene in wheat

**DOI:** 10.1073/pnas.2108808119

**Published:** 2022-07-20

**Authors:** Marion C. Müller, Lukas Kunz, Seraina Schudel, Aaron W. Lawson, Sandrine Kammerecker, Jonatan Isaksson, Michele Wyler, Johannes Graf, Alexandros G. Sotiropoulos, Coraline R. Praz, Beatrice Manser, Thomas Wicker, Salim Bourras, Beat Keller

**Affiliations:** ^a^Department of Plant and Microbial Biology, University of Zurich, 8008 Zurich, Switzerland;; ^b^Department of Plant Microbe Interactions, Max Planck Institute for Plant Breeding Research, 50829 Cologne, Germany

**Keywords:** wheat, powdery mildew, resistance introgression, gene conversion, avirulence gene

## Abstract

Introgressions of chromosomal segments from related species into wheat are important sources of resistance against fungal diseases. The durability and effectiveness of introgressed resistance genes upon agricultural deployment is highly variable—a phenomenon that remains poorly understood, as the corresponding fungal avirulence genes are largely unknown. Until its breakdown, the *Pm17* resistance gene introgressed from rye to wheat provided broad resistance against powdery mildew (*Blumeria graminis*). Here, we used quantitative trait locus (QTL) mapping to identify the corresponding wheat mildew avirulence effector *AvrPm17*. It is encoded by two paralogous genes that exhibit signatures of reoccurring gene conversion events and are members of a mildew sublineage specific effector cluster. Extensive haplovariant mining in wheat mildew and related sublineages identified several ancient virulent *AvrPm17* variants that were present as standing genetic variation in wheat powdery mildew prior to the *Pm17* introgression, thereby paving the way for the rapid breakdown of the *Pm17* resistance. QTL mapping in mildew identified a second genetic component likely corresponding to an additional resistance gene present on the 1AL.1RS translocation carrying *Pm17.* This gene remained previously undetected due to suppressed recombination within the introgressed rye chromosomal segment. We conclude that the initial effectiveness of 1AL.1RS was based on simultaneous introgression of two genetically linked resistance genes. Our results demonstrate the relevance of pathogen-based genetic approaches to disentangling complex resistance loci in wheat. We propose that identification and monitoring of avirulence gene diversity in pathogen populations become an integral part of introgression breeding to ensure effective and durable resistance in wheat.

## Significance

Domesticated and wild wheat relatives provide an important source of new immune receptors for wheat resistance breeding against fungal pathogens. The durability of these resistance genes is variable and difficult to predict, yet it is crucial for effective resistance breeding. We identified a fungal effector protein recognized by an immune receptor introgressed from rye to wheat. We found that variants of the effector allowing the fungus to overcome the resistance are ancient. They were already present in the wheat powdery mildew gene pool before the introgression of the immune receptor and are therefore responsible for the rapid resistance breakdown. Our study demonstrates that the effort to identify durable resistance genes cannot be dissociated from studies of pathogen avirulence genes.

Wheat is the most widely cultivated food crop and is susceptible to a number of fungal diseases. For more than a century, breeding for genetically resistant cultivars that can durably withstand disease has been one of the most important approaches for sustainable wheat production globally. Introgressions of chromosomal segments from closely related wild grasses such as *Aegilops* or *Agropyron* species (1, 2) and other crop species such as rye (*Secale cereale*) have been highly valuable sources of new resistance gene specificities (3). Specifically, the 1BL.1RS or 1AL.1RS translocations of the rye chromosome 1R introgressed into hexaploid (AABBDD) wheat (reviewed in ref. 4) were of great relevance for wheat resistance breeding. Genes present on these translocations are widely used in wheat breeding and confer resistance to leaf rust (*Lr26*), stripe rust (*Yr9*), stem rust (*Sr31*, *Sr50/SrR*, *Sr1R^Amigo^*), and powdery mildew (the allelic *Pm8/Pm17* pair) (5, 6).

It has been proposed that introgressed resistance genes provide more effective and potentially more durable resistance, since pathogens specialized on wheat have previously not been exposed and therefore have not adapted to the resistance specificities that evolved in other species (7). This is exemplified by the rye *Sr31* gene, which was deployed worldwide. It provided effective and broad resistance against *Puccinia graminis* f. sp. *tritici*, the causal agent of wheat stem rust for over 30 y before being overcome by the virulent African strain Ug99 (8), demonstrating both the huge benefit of an introgressed rye gene as well as the constant need for new broadly active resistance genes (9). In contrast to *Sr31* and the general hypothesis, many introgressed resistance genes were overcome quickly by wheat pathogens (10). For example, the rye introgressions with *Pm8* and *Pm17* became ineffective against wheat powdery mildew *Blumeria graminis* f. sp. *tritici* (*B.g. tritici*) within a few years after their deployment in large-scale agricultural settings (11–14). Thus, it remains one of the most pressing questions in the field of plant breeding research why a few introgressed genes such as *Sr31* remained effective over a long timeframe and despite worldwide deployment, whereas others are overcome quickly (7).

The allelic *Pm8* and *Pm17* genes encode for nucleotide-binding leucine-rich repeat (NLR) proteins that were introgressed into wheat from ‘Petkus’ and ‘Insave’ rye cultivars, respectively (15, 16). It was demonstrated that both genes represent rye homologs of the wheat *Pm3* resistance gene (15, 16), which encodes for a high number of different NLR alleles that confer race-specific resistance against wheat powdery mildew through recognition of mildew encoded avirulence proteins (17–19).

In wheat powdery mildew, recent studies using map-based cloning, genome-wide association studies, and effector benchmarking approaches have identified several avirulence genes, among them *AvrPm3^a2/f2^*, *AvrPm3^b2/c2^*, and *AvrPm3^d3^* recognized by *Pm3a/Pm3f*, *Pm3b/Pm3c*, and *Pm3d*, respectively (17, 18). Sequence analysis of wheat mildew *Avr* genes revealed that they all encode small secreted candidate effector proteins (17, 18, 20, 21) and exhibit high levels of sequence variation on a population level, including the independent evolution of numerous gain-of-virulence alleles by diverse molecular mechanisms (17, 18, 20, 22). The identification and functional characterization of mildew avirulence genes has therefore significantly broadened our understanding of race-specific resistance and resistance gene breakdown in the wheat–mildew pathosystem.

Grass powdery mildews exist in many sublineages also called formae speciales (f. sp.) that are highly host specific, such as mildew on wheat (*B.g. tritici*), rye (*B.g. secalis*), or the wheat/rye hybrid triticale (*B.g. triticale*) which emerged recently and was attributed to a hybridization event between wheat and rye mildew sublineages (23, 24). Due to the strict host barrier, it is assumed that nonadapted mildew sublineages have not been exposed to NLR resistance specificities of an incompatible host and therefore have not evolved to evade recognition. Indeed, several *Pm3* alleles have been found to contribute to nonhost resistance through recognition of conserved avirulence effectors in nonadapted mildew sublineages such as *B.g. secalis* (18). Given these observations, the rapid breakdown of *Pm8* and *Pm17* resistance after introgression into wheat remains puzzling and provides an opportunity to study evolutionary dynamics of wheat mildew in the context of introgression breeding. The *Pm17* introgression is especially suited for this purpose since the associated 1AL.1RS translocation, first described in 1976 in Oklahoma (25), was not used before the end of the 20th century, and has been deployed in large-scale agricultural setting only in the beginning of the 21th century in the United States, where it provided resistance against wheat mildew in bread wheat (26, 27). In contrast, the deployment in other wheat growing areas globally started only after the year 2000 (11, 27–29), and breakdown of *Pm17* resistance was generally observed within few years and has been well documented in several wheat growing regions such as the United States, China, and Switzerland (11, 13, 27, 30).

In this study, we report the molecular basis underlying the resistance breakdown of the introgressed *Pm17* gene in wheat. Using quantitative trait locus (QTL) mapping in a biparental mildew population, we demonstrate that the corresponding avirulence effector *AvrPm17* is encoded by a paralogous effector gene pair, residing in a dynamic effector cluster, specific to the wheat and rye mildew sublineages. Moreover, we describe the identification of numerous ancient virulence alleles of the *AvrPm17* gene that have been present as standing genetic variation in *B.g. tritici* even before the introgression of *Pm17* into the wheat breeding pool. Lastly, we provide genetic evidence for the existence of a so far unidentified resistance gene against wheat mildew that was cointrogressed with *Pm17* from rye, which could be revealed through careful dissection of resistance specificities based on genetic studies in the pathogen.

## Results

### QTL Mapping Identifies a Single Avirulence Locus for *Pm17* in Wheat Powdery Mildew.

**Fig. 1.**
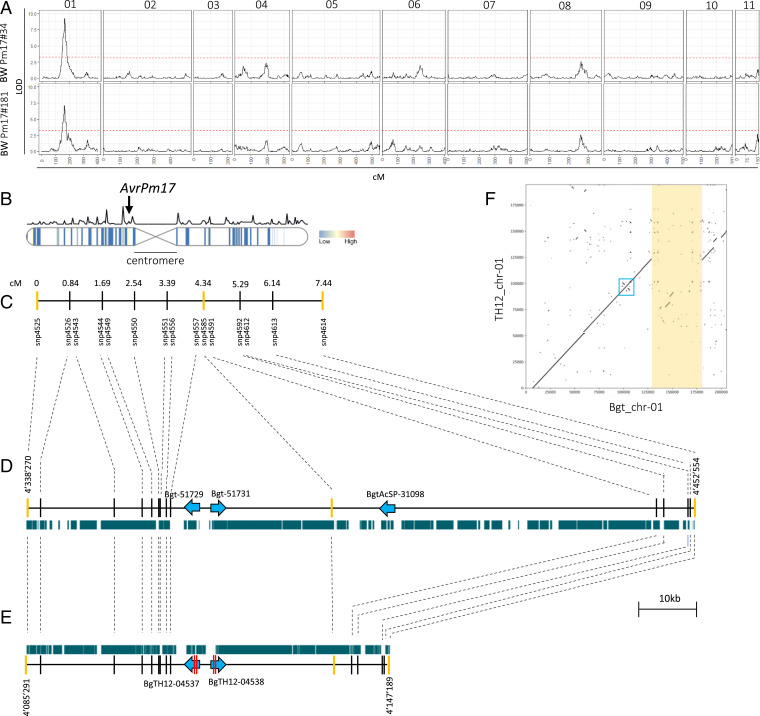
Avirulence on wheat genotypes with *Pm17* is controlled by a single locus in a biparental mapping population between *B.g. tritici* Bgt_96224 × *B.g. triticale* THUN-12. (*A*) Single interval QTL mapping of 55 progeny of the cross Bgt_96224 × THUN-12 on two transgenic lines expressing *Pm17*-HA under control of the maize ubiquitin promoter (Ubi) promoter. The genetic map of Bgt_96224 × THUN-12 based on 119,023 markers was reprinted from ref. 31, which is licensed under CC BY 4.0, and contains 11 linkage groups that correspond to the 11 chromosomes of *B.g. tritici* Bgt_96224 and *B.g. triticale* THUN-12 (31, 32). The significance level of the LOD (logarithm of the odds) value was determined using 1,000 permutations and is indicated by a red line. (*B*) Location of the QTLs identified in the pericentromeric region of chromosome 1 of *B.g. tritici* Bgt_96224. The centromeric region is indicated. Vertical bars indicate effector gene density in 50-kb windows following a gradient indicated in the color key. The line above the chromosome indicates the recombination rate in cM/50 kb as described in ref. 31. The remaining 10 chromosomes are depicted in *SI Appendix*, Fig. S2. (*C*) Informative markers in the 7.44-cM genetic CI (1.5 LOD) and their cM position relative to the left flanking marker. Flanking markers and the best associated marker of the QTL are depicted in yellow. (*D* and *E*) Physical interval underlying the genetic interval in *B.g. tritici* Bgt_96224 (*D*) and *B.g. triticale* THUN-12 (*E*). Gene and gene orientation are indicated with blue arrows (gene length not drawn to scale). Nonsynonymous SNPs in THUN-12 versus isolate Bgt_96224 are indicated by a red bar within the gene. Green bars indicates the presence of transposable elements in the interval. (*F*) Alignment showing the region flanking 100 kb up and downstream of the *AvrPm17* locus in the reference assemblies of *B.g. tritici* Bgt_96224 and *B.g. triticale* THUN-12. The location of the paralogous effectors *BgTH12-04537/BgTH12-04538* and *Bgt-51729/Bgt-51731* is indicated by a blue box. The 50-kb deletion in THUN-12 compared with Bgt_96224 is highlighted in yellow.

To understand the breakdown of the rye NLR *Pm17* in wheat, we aimed at identifying its corresponding avirulence gene by taking advantage of the recent cloning of *Pm17* and its validation in transgenic wheat lines (16). We used a preexisting, sequenced F1 mapping population derived from a cross of the avirulent *B.g. triticale* isolate THUN-12 and *B.g. tritici* isolate Bgt_96224, which exhibits a virulent phenotype on the independent transgenic lines Pm17#34 and Pm17#181 (*SI Appendix*, Fig. S1 *A* and *B*) (31). A single interval QTL mapping approach using 55 randomly selected progeny of the Bgt_96224 × THUN-12 cross identified a single locus on chromosome 1 at an identical map position (164.8 centimorgan [cM]) with highly significant logarithm of the odds (LOD) scores of 9.2 for wheat genotype Pm17#34 and 7.0 for Pm17#181, respectively (Fig. 1 *A*–*C* and *SI Appendix*, Fig. S1 *C* and *D* and Table S1). The pericentromeric location of the mapped *AvrPm17* locus contrasts with the location of previously identified wheat mildew avirulence genes that tend to reside near the telomeric region or on the chromosome arms (Fig. 1*B* and *SI Appendix*, Fig. S2; 17, 18, 20).

**Fig. 2.**
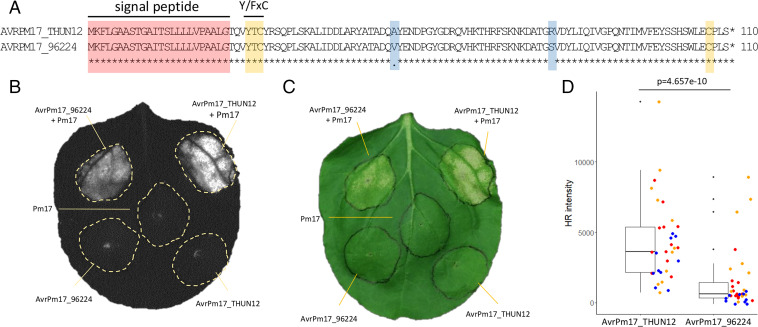
Functional validation of *AvrPm17* in *N. benthamiana.* (*A*) Protein alignment of the AVRPM17 candidate in THUN-12 and Bgt_96224. The sequence corresponding to the predicted signal peptide, the Y/FxC motif and the C-terminal cysteine residue are highlighted in red and yellow, respectively. Polymorphic amino acid residues between Bgt_96224 and THUN-12 are highlighted in blue. (*B* and *C*) Coexpression of *Pm17*-HA and *AvrPm17*_THUN12 (*BgTH12-04537/BgTH12-04538*) and *AvrPm17*_96224 (*Bgt-51729/Bgt-51731*) by transient *Agrobacterium*-mediated expression in *N. benthamiana,* imaged by the Fusion FX imager system (*B*) or a conventional camera (*C*) at 5 dpi. Coinfiltrations were done at a ratio of 1:4 R:Avr. Coexpression of *Pm17* with *AvrPm17_THUN-12* and *AvrPm17_96624* induced HR in *n* = 18 leaves in three independent experiments. No HR was observed when *AvrPm17_THUN-12*, *AvrPm17_96224*, or *Pm17* was expressed alone (*n* = 18). (*D*) Difference in HR induction between the two AVRPM17 variants AVRPM17_THUN12 and AVRPM17_96224 infiltrated in a ratio of R:Avr of 1:1. The *y-*axis represents quantitative measurement of HR based on the Fusion FX imager system. Individual datapoints are color coded based on three independent experiments with at least *n* = 8 leaves per experiment. The *P* value of the paired Wilcoxon-ranked sum test is indicated above the panel.

To identify *AvrPm17* candidate genes, we analyzed the physical region underlying the QTL (with a confidence interval [CI] of 1.5 LOD) on chromosome 1 in the chromosome-scale assemblies of the parental isolates Bgt_96224 and THUN-12 (31, 32). The genetic CI corresponded to a 61.8-kb region in the assembly of THUN-12 and a much larger region of 114.3 kb in the assembly of Bgt_96224. This striking difference in size is explained by a large 50-kb deletion in the THUN-12 genome (Fig. 1 *D*–*F*). The interval in the *Pm17* avirulent isolate THUN-12 only encodes the paralogous effector gene pair *BgTH12-04537* and *BgTH12-04538* (Fig. 1*E*). The two effector genes encode for identical proteins and differ by two synonymous single nucleotide polymorphisms (SNPs). The two gene copies are encoded by two inverted duplicated segments of 2,300 bp separated by a 4,769-bp intergenic region (Fig. 1*F*). The gene duplication is also present in the corresponding region of the virulent parent Bgt_96224 (Fig. 1*F* and *SI Appendix*, Table S2). There, the duplicated effector genes *Bgt-51729* and *Bgt-51731* are identical and encode for proteins that each carry two amino acid changes (A53V, R80S) compared with BgTH12-04537 and BgTH12-04538, respectively (Figs. 1 *D* and *E* and 2*A* and *SI Appendix*, Fig. S3). The interval in the Bgt_96224 genome encodes an additional effector gene, *BgtAcSP-31098*, that lies within the 50-kb deleted region in THUN-12. In the absence of additional genes in the locus of the avirulent parent THUN-12, we predicted that *BgTH12-04537* and *BgTH12-04538* encode for *AvrPm17*. Using RNA-sequencing data from the parental isolates, we found that *BgTH12-04537/BgTH12-04538* and *Bgt-51729/Bgt-51731* are highly expressed at early stages of infection, corresponding to the establishment of the haustorial feeding structure at 2 d post infection (dpi), reminiscent of other wheat mildew *Avr* genes (*SI Appendix*, Figs. S4 and S5). The *AvrPm17* candidates are not differentially expressed between the two isolates (logFC < 1.5; *SI Appendix*, Fig. S4), therefore indicating that the amino acid polymorphisms observed between Bgt_96224 and THUN-12 must account for the difference in phenotype.

A previous study found that the hybrid genome of *B.g. triticale* isolates consists of distinct genomic segments inherited from either wheat or rye mildew (23). Due to the rye origin of the *Pm17* resistance gene, the origin of the avirulence locus in triticale mildew THUN-12 is of special interest. Following the approach of Menardo et al. (23) based on the analysis of fixed polymorphism between wheat and rye mildew, we found that the physical region underlying the *AvrPm17* QTL in THUN-12 is a segment inherited from wheat mildew (*SI Appendix*, Fig. S6 *A*–*C*). This indicates that the rye *Pm17* gene recognizes an avirulence component originating from the nonadapted wheat powdery mildew donor, and not from the adapted rye mildew, in the triticale mildew hybrid.

### Functional Validation of *AvrPm17*.

To functionally validate *AvrPm17*, we transiently coexpressed the *BgTH12-04537/BgTH12-04538* Avr candidate with *Pm17*-HA in *Nicotiana benthamiana* by *Agrobacterium tumefaciens*–mediated transient overexpression (18, 20). All effector constructs were expressed without the signal peptide and codon optimized for expression in *N. benthamiana* to ensure optimal translation in planta. *BgTH12-04537/BgTH12-04538* elicited a strong hypersensitive response (HR) upon coexpression with Pm17-HA but not when expressed alone, confirming that these paralogous effector genes are *AvrPm17* (Fig. 2 *B* and *C*). Coexpression of *AvrPm17_THUN12* with either the *Pm8* gene from rye or the *Pm17* orthologs from wheat (*Pm3a-f*, *Pm3CS*) did not result in a HR in *N. benthamiana* (*SI Appendix*, Fig. S7), demonstrating the specificity of *AvrPm17_THUN-12* recognition by *Pm17*.

Interestingly, coexpression of *AvrPm17_96224* with *Pm17* also resulted in a HR (Fig. 2 *B* and *C*). The extent of cell death was, however, significantly reduced compared with the *AvrPm17_THUN12* variant (paired Wilcoxon rank test *P* = 4.657e-10) (Fig. 2*D*). We therefore concluded that *Pm17* can weakly recognize *AvrPm17_96224*, at least in a heterologous overexpression system. To address the question of whether the weak recognition of *AvrPm17_96224* translates into phenotypes on *Pm17* wheat, we made use of the above-mentioned transgenic lines Pm17#181 and Pm17#34 which were previously shown to exhibit differences in PM17 protein abundance, with Pm17#181 representing the stronger line (16). Consistent with a prediction of a quantitative difference in AVR recognition, we observed reduced mildew leaf coverage upon infection of both transgenic *Pm17* wheat lines with isolate Bgt_96224 and with progeny of the Bgt_96224 × THUN-12 cross carrying the *AvrPm17_96224* haplotype (*SI Appendix*, Fig. S1 *A*–*D*), thus indicating that a residual recognition of *AvrPm17_96224* reduces disease severity quantitatively. In contrast, the recognition of the *AvrPm17_THUN12* haplotype conferred complete disease resistance in both transgenic lines (*SI Appendix*, Fig. S1 *A*–*D*).

We therefore also analyzed differences in AVR protein abundance using C-terminal FLAG epitope-tagged AVRPM17 variants from Bgt_96224 and THUN-12. The presence of the FLAG epitope partially interfered with *AvrPm17* recognition since tagged versions exhibited significantly reduced HR levels when coexpressed with *Pm17*; however, the specificity was not affected (*SI Appendix*, Fig. S8 *A*–*D*). Both AVRPM17-FLAG variants as well as PM17-HA were detectable on a Western blot (*SI Appendix*, Fig. S8 *E* and *F*). Interestingly, expression of AVRPM17_THUN12 in *N. benthamiana* resulted in higher protein abundance than for the weakly recognized AVRPM17_96224 variant (*SI Appendix*, Fig. S8*F*). This suggests that the amino acid polymorphisms between Bgt_96224 and THUN-12 affect protein levels of AVRPM17, at least in the heterologous *Nicotiana* system. Similar observations were recently described for *AvrPm3^a2/f2^*, where polymorphism in the AVR was found to affect the protein amount and thereby directly influence recognition by *Pm3a* (33).

AVRPM17 is part of effector family E003, the second largest effector family found in *B. graminis* (31). The family is composed of small proteins of approximately 110 amino acids that contain a predicted signal peptide, an N-terminal Y/FxC motif followed by a stretch of alternating hydrophobic residues, as well as a conserved carboxyl-terminal cysteine (*SI Appendix*, Fig. S9). These features have been described for numerous *Blumeria* effectors, including all functionally characterized AVR proteins in wheat mildew (18, 20, 21). Using an in silico modeling approach based on IntFOLD5.0, we found that AVRPM17 is predicted to exhibit a ribonuclease-fold (*P* = 1.145E-4), consisting of a single α-helix and three β-strands (*SI Appendix*, Fig. S10 *A* and *B*). A similar ribonuclease-fold was experimentally determined by crystallization for the barley powdery mildew (*Blumeria graminis* f. sp. *hordei*) effector BEC1054 (34) and proposed for AVRA7, the avirulence gene of the barley NLR Mla7 (35). *Avra7* is part of the *AvrPm17* gene family (E003) (*SI Appendix*, Fig. S11), suggesting that the ribonuclease-fold is conserved within the effector family. The particular arrangement of α-helix followed by multiple β-strands has been predicted for other AVR proteins in wheat mildew. Most importantly, such a pattern was described for the entire effector families E008, E018, and E034 encoding *AvrPm3^a2/f2^*, *AvrPm3^b2/c2^*, and *AvrPm3^d3^*, respectively (18). We therefore hypothesize that *AvrPm17* and the *AvrPm3*s encode for structurally similar proteins, despite little similarity on the primary amino acid sequences (*SI Appendix*, Fig. S12), and that the wheat *Pm3* allelic series and its rye ortholog *Pm17* recognize structurally related effectors.

### *AvrPm17* Is Encoded in a Mildew Sublineage-Specific Effector Cluster and Exhibits Signs of Reoccurring Gene Conversion Events.

**Fig. 3.**
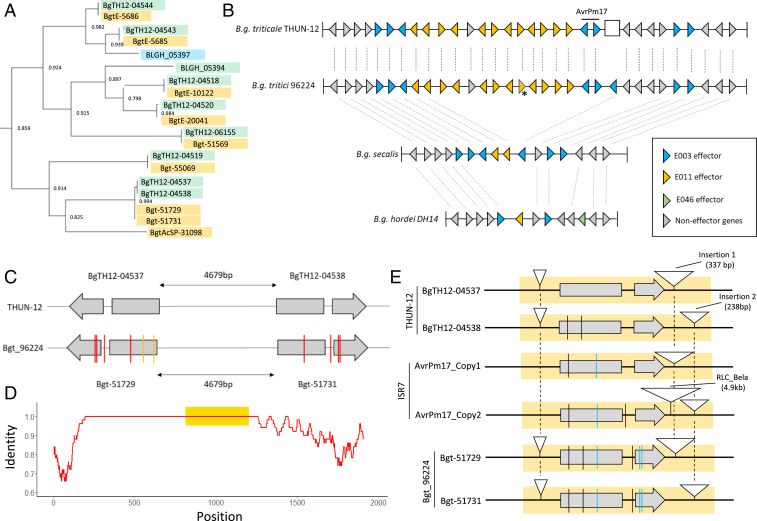
*AvrPm17* is a member of a highly expanded effector gene cluster. (*A*) Phylogenetic relationship of the AVRPM17 effector family. *A* shows a subsection of the phylogenetic tree based on protein sequences of E003 effector family members of *B.g. tritici* (69 members), *B.g.* *triticale* (70 members), and *B.g. hordei* (59 members). The full tree can be found in *SI Appendix*, Fig. S11. Effector family members are highlighted as follows: members in *B.g. tritici* in yellow, members in *B.g. triticale* in green, and members in *B.g. hordei* in blue. For each branch, the local support values calculated with the Shimodaira–Hasegawa test are indicated. (*B*) Schematic representation of the *AvrPm17* effector cluster in the high-quality genomes of *B.g. triticale* THUN-12, *B.g. tritici* Bgt_96224, and *B.g. hordei* DH14. In the absence of a high-quality genome assembly for *B.g. secalis*, the presence/absence of genes was estimated by coverage analysis based on mappings of five resequenced isolates. Genes that are present in at least one *B.g. secalis* isolate were considered as present. Genes and their orientation are indicated by triangles. The white rectangle in the *B.g. triticale* THUN-12 assembly indicates the position of the 50-kb deletion presented in Fig. 1*F*. The gene marked with an asterisk represents a collapsed gene duplication in the *B.g. tritici* Bgt_96224 assembly that was resolved in the *B.g. triticale* THUN-12 genome assembly. The syntenic relationship is indicated by dashed lines. The figure is not drawn to scale. (*C–E*) The *AvrPm17* gene copies have evolved through gene conversion. (*C*) Analysis of SNPs in the *AvrPm17* gene copies in the two parental isolates. SNPs in Bgt_96224 are shown in comparison with THUN-12. The *AvrPm17* genes are represented schematically with gray boxes representing the two exons. The transcriptional orientation is indicated by the direction of the arrowhead in the second exon. SNPs are indicated in the coding sequences and the intron. Red bars represent SNPs that are shared between the two gene copies in Bgt_96224 and yellow bars indicate SNPs that are present in only one copy. (*D*) Visual representation of the duplication of *AvrPm17* in isolate Bgt_96224. To allow alignment of the two sequences, the insertions in the downstream region of the two genes were spliced out. The *x*-axis shows the alignment position, while the *y*-axis shows the sequence identity calculated in 50-bp sliding windows. The position of the *AvrPm17* gene is highlighted by a yellow box. (*E*) Schematic representation of the duplicated segments (as yellow boxes) containing *AvrPm17* genes (indicated by gray arrows) in the three high-quality genomes of THUN-12, ISR7, and Bgt_96224. SNPs are indicated compared with *BgTH12-04537* as follows: blue indicates nonsynonymous SNPs, and black indicates synonymous SNPs or SNPs in the intron. White triangles indicate insertions.

Candidate effector genes in wheat and barley powdery mildew have been grouped into 235 families based on sequence similarity (31). *AvrPm17* belongs to family E003, which is represented with 69 members in *B.g. tritici* (isolate Bgt_96224), 70 members in *B.g. triticale* (isolate THUN-12), and 59 members in *B.g. hordei* (isolate *DH14*) (31, 35). E003 is physically organized in gene clusters distributed over 7 of the 11 chromosomes of wheat and triticale mildew (*SI Appendix*, Fig. S11). Family members encoded in the same chromosomal location form phylogenetically related clades, consistent with the previously proposed expansion mechanism of effector genes through local duplication (*SI Appendix*, Fig. S11; ref. 22). Interestingly, the *AvrPm17* clade is encoded in a gene cluster that spans more than 1.3 Mb on chromosome 1 and contains seven and eight members in triticale and wheat mildew, respectively, as well as a solitary family member on chromosome 8 (Fig. 3 *A* and *B* and *SI Appendix*, Fig. S11). The locus also harbors an additional effector cluster from family E011, consisting of 11 members, that has expanded within the E003 cluster (Fig. 3*B*). To study the evolutionary history of the *AvrPm17* clade, we identified the region corresponding to the *AvrPm17* cluster on scaffold_27 in barley mildew isolate DH14 based on conserved syntenic flanking genes. Strikingly, the region in DH14 is only 200 kb in size and harbors only two E003 family members of the *AvrPm17* clade as well as a single effector from family E011 (Fig. 3*B*). This indicates that both effector clades have been significantly expanded in wheat mildew after the divergence from the barley mildew lineage (Fig. 3*B*). Using resequencing data from five rye mildew strains, we could also show that rye mildew has six out of the eight E003 family members in the clade, whereas it lacks most of the E011 genes (Fig. 3*B*). These findings indicate that the E003 expansion has happened in a progenitor of rye/wheat mildew, whereas the E011 family expansion in this region is wheat mildew specific. Strikingly, rye mildew does not encode for an *AvrPm17* gene, consistent with the virulent phenotype of the five rye mildew isolates on the *Pm17*-donor line ‘Insave’ (*SI Appendix*, Fig. S13). This might indicate that *AvrPm17* was lost in rye mildew possibly due to the selection pressure imposed by the *Pm17* gene in the rye gene pool (*SI Appendix*, Table S3).

Given the highly dynamic genomic context of the *AvrPm17* locus, it is striking that the avirulent AVRPM17_THUN12 variant and the partial gain-of-virulence variant AVPM17_96224 are encoded by near identical (*BgTH12-04537/BgTH12-04538,* two synonymous SNPs) or identical (*Bgt-51729/Bgt-51731*) paralogous gene copies within the isolates THUN-12 and Bgt_96224, respectively (*SI Appendix*, Fig. S14). Most importantly, the three nonsynonymous SNPs (two affecting the same codon) that differentiate *AvrPm17_96224* from the avirulent *AvrPm17_THUN12* are identical in both genes *Bgt-51729* and *Bgt-51731* (Fig. 3*C*). Congruently there is an identical SNP in the intron of *Bgt-51729* and *Bgt-51731* when compared with both genes in THUN-12 (*BgTH12-04537/BgTH12-04538*). Strikingly, the *AvrPm17* locus in the newly sequenced genome assembly of another *B.g. tritici* isolate ISR7 also contains two *AvrPm17* gene copies that are encoded in the same inverted direction (*SI Appendix*, Fig. S15*A*). Both *AvrPm17*_*ISR7* gene copies also encode again for identical proteins that share the A53V substitution with AVRPM17_96224. There are three potential explanations for how paralogous genes copies in three isolates are more similar to each other than to their respective ortholog: 1) independent accumulation of identical mutations in both genes, 2) recurrent gene duplication, and 3) transfer of mutation from one gene to the other by gene conversion. It is highly unlikely that the exact same four mutations have occurred independently in both genes. Furthermore, the duplicated region in Bgt_96224, THUN-12, and ISR7 is identical in size and position and therefore must have occurred in the ancestor of the three isolates, predating the accumulation of the mutations differentiating the *AvrPm17* genes (Fig. 3*C* and *SI Appendix*, Fig. S15*A*). Consistent with the hypothesis of a more ancient duplication event, we found that flanking regions of the duplicated segment are significantly more divergent (Fig. 3*D* and *SI Appendix*, Fig. S16). Importantly, the duplicated segments have acquired insertions in the noncoding region (Fig. 3*E* and *SI Appendix*, Fig, S15*A*). These insertions are specific for one copy of the duplicated segments and are present and identical in all three haplotypes (Fig. 3*E* and *SI Appendix*, Figs. S15 and S17 and Text 1). The presence of these insertions corroborates that *AvrPm17_96224*, *AvrPm17_THUN-12*, and *AvrPm17_ISR7* gene copies originate from the same gene duplication event and not from independent duplications. Therefore, the nucleotide polymorphisms defining the differences between *AvrPm17_THUN12*, *AvrPm17_96224*, and *AvrPm17_ISR7* have most probably occurred in one gene copy and were then transferred to its duplicate by gene conversion (for further details, see *SI Appendix*, Text 1). We propose that gene conversion event(s) contribute to the evolutionary potential of *AvrPm17* as an efficient way to transfer beneficial mutations to both gene copies.

### Virulent *AvrPm17* Haplovariants Are Ancient and Predate *Pm17* Introgression into Wheat.

**Fig. 4.**
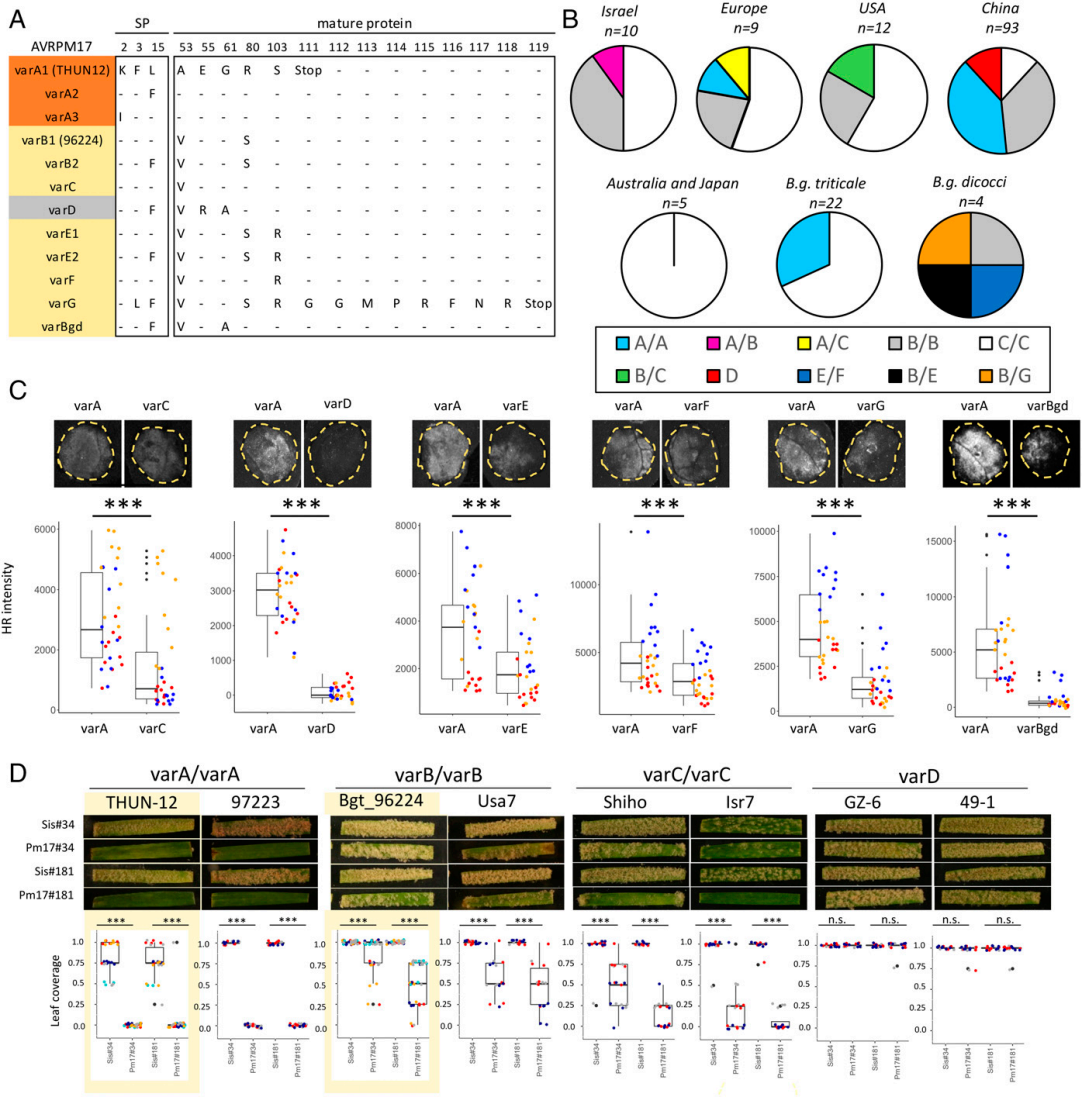
Diversity analysis of AVRPM17 in different formae speciales. (*A*) Protein variants found in a global mildew population of 156 isolates (*B.g. tritici*, *B.g. triticale*, *B.g. dicocci*, *B.g. dactylidis*). Colors indicate differences in recognition strength determined by transient coexpression in *N. benthamiana*. Orange indicates the avirulence allele varA and haplotypes that are recognized to a comparable extent. Yellow indicates variants that are recognized significantly weaker than the avirulent allele *AvrPm17*_varA and therefore represent partial gain-of-virulence alleles. The only full loss-of-recognition variant is indicated in gray. (*B*) Distribution of haplotypes in global *B.g. tritici* (Israel, Europe, United States, China, Australia, and Japan), *B.g. triticale*, and *B.g. dicocci* populations. (*C*) Recognition strength of AVRPM17 variants (depicted in *A*) in *N. benthamiana* compared with AVRPM17_varA. Infiltrations were performed at R: Avr ratios of 1:1 and significance was assessed using a paired Wilcoxon rank sum test. Significance levels are indicated above the boxplots as follows: ***P < 0.001. The *y*-axis represents quantitative measurement of HR based on the Fusion FX imager system. Individual datapoints are color coded based on three independent experiments with at least *n* = 8 leaves per experiment. (*D*) Phenotypes of representative isolates carrying varA/varA, varB/varB, varC/varC, and varD haplotypes on two *Pm17* transgenic lines and the corresponding sister lines. Leaf coverage of individual leaf segments was scored according to the following scale: avirulent = 0, avirulent/intermediate = 0.25, intermediate = 0.5, intermediate/virulent = 0.75, and virulent = 1. Boxplots represent data of at least three independent infection experiments (five for Bgt_96224 and THUN12) with at least three leaf segments scored per experiment. Boxplots and pictures surrounded by a yellow box indicate data on the reference isolates that are also represented in *SI Appendix*, Fig. S1 *A* and *B* and are included here for the purpose of comparison with the other isolates. Dots represent individual leaf segments and are color coded according to the infection experiment. Significance was assessed using the exact Wilcoxon rank test, and significance levels are indicated above the boxplots as follows: ***P < 0.001; n.s., not significant.

A haplotype mining approach in a diversity panel of 151 resequenced isolates of wheat mildew (129 isolates) and triticale mildew (22 isolates) for *AvrPm17* revealed there are three dominant AVRPM17 variants in the gene pool (Fig. 4 *A* and *B*). Two of these are the above-described avirulent AVPM17_THUN12 (varA) and the weakly recognized AVPM17_96224 (varB). The most frequent haplotype is varC (AVRPM17_ISR7) that contains a single amino acid polymorphism (A53V) and induces weaker HR in the *Nicotiana* coexpression assays as compared with the functional varA found in THUN-12 (Fig. 4*C*). In addition, 11 isolates originating from China encode for the only complete loss-of-recognition haplotype found (varD) with three amino acid changes (A53V, E55R, G61A) compared with varA (Fig. 4 *A*–*C* and *SI Appendix*, Fig. S18). Importantly, all *AvrPm17* variants were found to be expressed during early infection of wheat (i.e., the haustorial stage) in isolates containing each of the four haplovariants varA to varD (*SI Appendix*, Fig. S19).

Based on sequencing coverage, we estimated that the majority of isolates encoding varA, varB, or varC contain two *AvrPm17* copies, whereas isolates that encode varD encode for a single *AvrPm17* gene (*SI Appendix*, Fig. S20 and Dataset S3). In addition, three isolates likely encode more than three *AvrPm17* copies (*SI Appendix*, Fig. S20). Strikingly, 97% of the isolates with two *AvrPm17* copies encode for two identical mature AVRPM17 proteins in one of the following combinations: varA/varA, varB/varB, and varC/varC (Fig. 4*B*). This supports the hypothesis that the *AvrPm17* gene copies are kept identical by recurring gene conversion events (for details, see *SI Appendix*, Text 2; Figs. S15–S17 and S21; and Table S4). A PCR-based *AvrPm17* locus dissection in 16 isolates representing the four most commonly found combinations varA/varA, varB/varB, varC/varC, or varD confirmed the presence and conserved position of the specific insertions that distinguish the two duplicated segments in all isolates containing two *AvrPm17* copies (*SI Appendix*, Fig. S15 *A*–*F*). This further corroborates that identical *AvrPm17* gene copies in these isolates did not evolve from independent duplications but are derived from the same ancient gene duplication.

In a next experiment, we analyzed the virulence phenotype of the above-mentioned 16 representative isolates covering the diversity of *AvrPm17* in wheat mildew (varA to varD), on *Pm17* transgenic wheat lines (Fig. 4*D* and *SI Appendix*, Table S5). Consistent with the strong HR induction of varA upon coexpression with *Pm17* in *N. benthamiana*, isolates encoding the varA/varA variant were completely avirulent on both transgenic lines (Fig. 4*D* and *SI Appendix*, Fig. S1 *A* and *B* and Table S5). In accordance with the absence of HR in *N. benthamiana*, isolates encoding varD displayed full virulence on the transgenic *Pm17* lines (Fig. 4*D* and *SI Appendix*, Fig. S18 and Table S5), demonstrating that the race specificity of the transgenic lines is retained despite *Pm17* overexpression. Infections of the *Pm17* transgenic lines with isolates containing the varB/varB or varC/varC variants resulted in a quantitative reduction of leaf coverage when compared with the corresponding sister lines (Fig. 4*D* and *SI Appendix*, Table S5). This reduction in infection success is consistent with the data from *N. benthamiana*, in which both the varB and varC variant induced an HR response that was however significantly weaker than by the avirulence variant varA (Figs. 2 *B*–*D* and 4*C* and *SI Appendix*, Fig. S18). To independently validate the observed quantitative differences in *Pm17*-mediated cell death, we measured wheat protoplast viability (i.e., cell death) upon cotransfection of *Pm17* with different *AvrPm17* variants. Cotransfection of *Pm17* with *AvrPm17*_varA strongly reduced protoplast viability compared with the nonrecognized varD (*SI Appendix*, Fig. S22*A*). In addition, we observed intermediate protoplast viability upon cotransfection of varB or varC with *Pm17* compared with the nonrecognized varD variant and recognized varA (*SI Appendix*, Fig. S22*A*), which is consistent with the intermediate reactions toward varB and varC observed in *N. benthamiana* (Fig. 4*C*) and on *Pm17* transgenic wheat lines (Fig. 4*D*). Taken together, the three assays (*N. benthamiana*, wheat protoplast, wheat phenotyping) consistently show the strongest resistance reaction for varA followed by varC and varB (varC > varB) and no effect for the nonrecognized varD (Fig. 4 *C* and *D* and *SI Appendix*, Fig. S22 *A* and *B*). These data indicate quantitative activation of *Pm17*-mediated resistance by different *AvrPm17* variants. Furthermore, these findings indicate that *AvrPm17*_varD and *AvrPm17*_varB/varC indeed represent virulence or partial virulence alleles, respectively, that are likely responsible for the resistance breakdown of the *Pm17* gene in wheat.

Both partially virulent variants varB and varC were present in all major subpopulations (i.e., China, Europe, Israel, United States) (Fig. 4*B*). Given their global distribution and considering that some of the isolates were collected already in the 1990s (Dataset S3), this suggests that the partially virulent *AvrPm17* variants varB and varC were present as standing genetic variation in the wheat mildew population before large-scale agricultural deployment of wheat varieties carrying the *Pm17* introgression at the beginning of the 21st century in the United States, and only subsequently in other regions of the world (27, 29). To further test this hypothesis, we extended our haplotype analysis to closely related formae speciales of *B.g. tritici*. Due to their distinct host range, *Blumeria graminis* f. sp. *dicocci*, a forma specialis sampled on wild tetraploid wheat, and *Blumeria graminis* f. sp. *dactylidis* infecting the wild grass *Dactylidis glomerata* (23, 36) are unlikely to have previously been exposed to the *Pm17* resistance gene. Strikingly, we found that three isolates of *B.g. dicocci* encode up to two copies of *AvrPm17_varB* (Fig. 4*B*). Furthermore, we found three additional haplovariants (varE to varG) specific to *B.g. diccoci* (Fig. 4 *A* and *B*). Coexpression of varE-G with *Pm17* in *N. benthamiana* resulted in significantly weaker HR responses compared with *AvrPm17_varA*, suggesting that these variants might also represent partially virulent alleles (Fig. 4*C* and *SI Appendix*, Fig. S18). This is consistent with the observation that these haplovariants share the A53V, R80S mutation (varE,G) or the A53V mutation (varF) with AVRPM17_varB (Fig. 4*A*). Since *B.g. dicocci* does not grow on most hexaploid wheat cultivars, including ‘Bobwhite’ (23), we could not test the contribution of the varE-G recognition to *Pm17* virulence. In *B.g. dactylidis*, represented by two isolates, we found an additional haplovariant *AvrPm17*_varBgd, which carries two substitutions (A53V and G61A) compared with AVRPM17_varA and is only very weakly recognized by *Pm17* in *N. benthamiana* (Fig. 4 *A* and *C*). Most importantly, these mutations are shared with the nonrecognized Chinese haplotype *AvrPm17_varD*, demonstrating that 1) the E55R substitution in the Chinese haplotype is the causative mutation leading to complete loss of recognition by *Pm17* (Fig. 4*C* and *SI Appendix*, Fig. S18) and 2) that part of the *AvrPm17* diversity found in wheat mildew is ancient and predates the split of *B.g. tritici* and *B.g. dactylidis*. Taken together, we found that a significant proportion of the *AvrPm17* sequence diversity found in *B.g. tritici*, including several gain-of-virulence mutations, is shared with its closely related formae speciales *B.g. dicocci* or *B.g. dactylidis*. Combined with the observation of a global distribution of partially virulent *AvrPm17* variants varB and varC and their presence in isolates collected before the deployment of *Pm17* wheat in agriculture, our findings strongly indicate that these *AvrPm17* gain-of-virulence mutations represent standing genetic variation in wheat mildew which predates the introgression of *Pm17* into wheat.

While the existence of numerous virulent or partially virulent *AvrPm17* haplotypes in the global mildew population prior to *Pm17* introgression might explain rapid *Pm17* resistance breakdown, this observation is hardly compatible with the initially described broad resistance phenotype exerted by the 1AL.1RS translocation. Based on these considerations, we therefore hypothesized the 1AL.1RS translocation to harbor a second mildew resistance gene in addition to *Pm17*.

### The 1RS.1AL Translocation Encodes for Two Powdery Mildew Resistance Specificities.

**Fig. 5.**
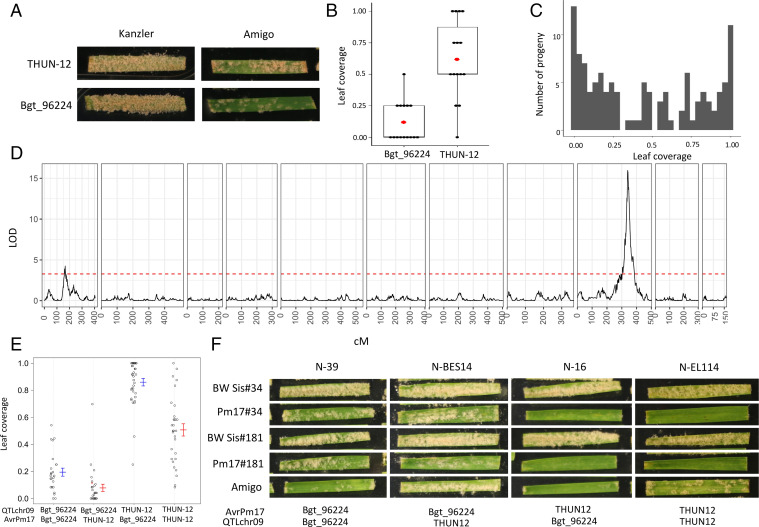
QTL mapping in the Bgt_96224 × THUN-12 F1 population on the wheat cultivar ‘Amigo’ carrying a 1RS.1AL translocation from ‘Insave’ rye including the *Pm17* resistance gene. (*A*) Representative photographs of phenotypes of the parental isolates Bgt_96224 and THUN-12 on ‘Amigo’ at 10 dpi. The susceptible wheat cultivar ‘Kanzler’ was used as an infection control. (*B*) Boxplot summarizing the phenotypes of Bgt_96224 and THUN-12 on ‘Amigo’. Leaf coverage of individual leaf segments was scored according to the following scale: avirulent = 0, avirulent/intermediate = 0.25, intermediate = 0.5, intermediate/virulent = 0.75, and virulent = 1 (*n* = 15, three independent experiments). (*C*) Distribution of phenotypes of the 117 progeny of the cross Bgt_96224 × THUN-12 on ‘Amigo’. Progeny phenotypes were scored as described in *B* and the average of at least six leaf segments for each progeny was plotted. (*D*) Single interval QTL mapping of Bgt_96224 × THUN-12 on cultivar ‘Amigo’. The black line indicates the LOD score of the association throughout the 11 chromosomes of wheat powdery mildew. The red line indicates the significance threshold determined by 1,000 permutations. (*E*) QTL effect plots summarizing phenotypes of the 117 progeny on ‘Amigo’. The phenotypes were plotted based on the genotypes of the best associated marker at the QTL location on chromosome 9 (QTLchr09) and chromosome 1 (*AvrPm17* locus). (*F*) Photographs of representative progeny of the cross Bgt_96224 × THUN-12 with different genotype combinations (see *E*) for the QTLs identified in *D*. Phenotypes on wheat cultivar ‘Amigo’ and the transgenic lines expressing *Pm17* at 10 dpi are shown.

To test for the predicted second resistance gene of the 1AL.1RS translocation, we characterized the genetic association of avirulence of the Bgt_96224 × THUN-12 mapping population on the original 1AL.1RS translocation line ‘Amigo’ (16). The *Pm17* avirulent isolate THUN-12 showed an intermediate phenotype on ‘Amigo’, demonstrating that recognition of *AvrPm17_varA* results in quantitative resistance in the presence of the endogenous *Pm17* gene (Fig. 5 *A* and *B*). In contrast, the *Pm17*-virulent isolate Bgt_96224 was avirulent on ‘Amigo’, indicating that 1) this isolate carries an additional avirulence component recognized by ‘Amigo’ and 2) the Bgt_96224 × THUN-12 biparental population is suited to validate the second resistance specificity in ‘Amigo’ (Fig. 5*C*). Consistent with our hypothesis, a QTL mapping analysis based on 117 progeny of Bgt_96224 × THUN12 identified two significant QTLs associated with the avirulence phenotype on cultivar ‘Amigo’ (for details, see Fig. 5*D* and *SI Appendix*, Text 3 and Table S6). One QTL on chromosome 1 corresponds to the *AvrPm17* locus, thereby verifying the activity of the *Pm17* gene in the original translocation line ‘Amigo’. In addition, we identified a highly significant QTL on chromosome 9 that was not detected in the QTL analysis on the transgenic *Pm17* lines (Figs. 1*A* and 5*D*), likely encoding the avirulence component recognized by the predicted second resistance gene of the 1AL.1RS translocation. The CI of the QTL on chromosome 9 encompasses 371 kb in the avirulent isolate Bgt_96224 and harbors a total of 16 effector genes, of which four are polymorphic compared with THUN-12 (*SI Appendix*, Table S7 and Fig. S23*A*). The corresponding genomic region in the hybrid *B.g. triticale* THUN-12 is again inherited from *B.g. tritici* (*SI Appendix*, Table S6). Upon coexpression with *Pm17* in *N. benthamiana*, none of the effector candidates encoded by isolate Bgt_96224 within the CI elicited a HR (*SI Appendix*, Fig. S23*B*). This finding demonstrates that the second QTL on chromosome 9 is independent of the *Pm17* resistance specificity. Most importantly, only progeny of the cross that carry the *AvrPm17_96224* haplovariant (*AvrPm17_varB*) and the THUN-12 genotype in the QTL on chromosome 9 are fully virulent (Fig. 5 *D* and *E*), further demonstrating that the simultaneous presence of both virulence alleles is necessary to overcome the resistance on ‘Amigo’.

We therefore conclude that the broad effectiveness of the 1AL.1RS translocation in providing resistance against wheat powdery mildew was based on two resistance gene specificities in the 1AL.1RS translocation. Since the *Pm17* resistance specificity has been attributed to a single locus based on segregation analysis, we hypothesize that the second locus is genetically linked on the 1AL.1RS region and has previously been genetically masked due to repressed recombination frequently associated with introgressed segments in wheat (37).

In summary, we here demonstrate that the *Pm17* introgression is genetically complex and that such complexity could only be revealed through accurate genetic dissection of the avirulence determinants in the pathogen distinguishing the two resistance specificities.

## Discussion

The recent identification of numerous *Avr* genes both in *B.g. tritici* and *B.g. hordei* has significantly advanced our understanding of NLR-mediated resistance in the cereal powdery mildew pathosystem (17, 18, 20–22, 35, 38). The functional cloning of *Avr* genes not only allowed molecular studies on recognition mechanisms (33, 35) but has also set the ground for genetic studies based on the natural diversity of avirulence components in local and global mildew collections. This has led to the discovery of numerous gain-of-virulence mechanisms exerted by *Blumeria* pathogens, including single amino acid polymorphisms, truncations, and deletions of *Avr* genes as well as a fungal encoded suppressor *SvrPm3* acting on *Pm3*-mediated resistance through masking of AVRPM3 recognition (17, 18, 35). These findings highlight the importance of genetic and genomic studies in fungal plant pathogens in order to understand the mechanisms of resistance breakdown and allow us to adapt current breeding approaches toward more durable deployment of resistance genes in cereal crops.

After cloning and functional characterization of so far 10 *Blumeria Avr* genes, several patterns emerged. *Blumeria* AVR effectors were found to be small proteins with a length of 102 to 130 amino acids and to contain an N-terminal signal peptide, a largely conserved Y/FxC motif, and a conserved cysteine residue toward the C terminus (17, 18, 20–22, 35, 38), while otherwise exhibiting highly divergent amino acid sequences. Furthermore, wheat mildew *Avr*s were consistently among the highest expressed genes within their effector gene family, indicating high abundance in host cells upon secretion presumably influencing the efficacy of their virulence function alongside NLR-mediated recognition in resistant cultivars (18). The newly identified *AvrPm17* exhibits all of the above-mentioned characteristics of *Blumeria* AVRs and therefore further corroborates the emerging patterns.

Despite showing little homology to proteins with a known function, more than a hundred *Blumeria* effectors are predicted to exhibit a ribonuclease-like fold (20, 34, 35, 39, 40). Notably, such a ribonuclease-like structure has recently been confirmed by protein crystallization of the barley powdery mildew effector BEC1054 (34). Similarly, despite highly divergent amino acid sequences, in silico protein modeling approaches predicted ribonuclease-like folds for most of the functionally verified avirulence proteins in barley and wheat mildew (20, 21, 35), including all AVRPM3 effectors (18) and AVRPM17 (this study). Based on the homology between rye *Pm17* and wheat *Pm3* NLRs combined with the predicted structural similarities of their corresponding AVR proteins, we propose a conserved recognition mechanism, likely leading to similar selection pressures acting on AVR genes for evasion of recognition.

Extensive haplovariant mining in a global wheat mildew collection for *AvrPm3^a2/f2^*, *AvrPm3^b2/c2^*, and *AvrPm3^d3^* revealed that virulent alleles were exclusively based on single amino acid polymorphisms (18, 22). Even though copy number variation was common, disruption or deletion of the avirulence gene, as observed for many other *AVR*s, has never been detected. In line with these findings, our haplovariant mining approach for the paralogous *AvrPm17* copies in a comparable wheat mildew diversity panel also failed to identify gene deletions or nonsense mutations and in return found four variants varA to varD, of which three represent partial or complete virulence alleles that are based on amino acid polymorphisms. In contrast, we found the *AvrPm17* genes to be absent in rye mildew, suggesting different gain-of-virulence mechanisms in these closely related mildew sublineages, likely due to differences in the exposure to the *Pm17* gene. Alternatively, the rye lineage might have never possessed an *AvrPm17* gene. Therefore, expanding the diversity panel of rye mildew isolates will be crucial to disentangle the evolutionary history of the *AvrPm17* in this mildew lineage. Such analyses will help to determine if *Pm17* in rye still provides race-specific resistance against certain rye mildew isolates.

Gene duplications in effector genes are common and considered advantageous for pathogens, as they allow the independent diversification of virulence factors (31). However, the presence of identical avirulence gene copies can represent a major liability, as gain-of-virulence mutations need to occur in both gene copies to effectively change the phenotypic outcome. This was described for wheat mildew *AvrPm3^d3^* in which gain-of-virulence mutations in one of the tandem duplicated gene copies was not sufficient to render the isolates virulent (18). Gene conversion, efficiently transferring beneficial mutations between gene copies, could provide pathogens with a molecular mechanism to mitigate the disadvantage of duplicated avirulence genes. Indeed, a case of gene conversion leading to gain of virulence was described for *Avr3c* in the oomycete *Phytophthora sojae* (41). Similarly, we found evidence for gene conversion to have occurred between the paralogous copies of *AvrPm17* (*SI Appendix*, Text 1 and 2). The high frequency of wheat mildew isolates encoding for varA/varA, varB/varB, or varC/varC genotypes furthermore indicates repeated gene conversion events between the two paralogs. Whether this phenomenon is dependent on inherent predisposition of the locus to nonallelic gene conversion to occur or whether it reflects the existence of an additional selection pressure linked to the virulence function of *AvrPm17* to maintain the sequences identical will be subject to further studies.

It was hypothesized that introgressed *R* resistance genes provide effective and durable resistance by recognizing an effector gene which, in the absence of previously acting diversifying selection, is largely conserved (7). The opportunity to test this hypothesis for a fungal pathogen in wheat arose with the recent cloning of the introgressed stem rust resistance genes *Sr35* [from *Triticum monococcum* (42)] and *Sr50* [from *S. cereale* (43)] and their corresponding avirulence genes *AvrSr35* and *AvrSr50* in *Puccinia graminis* f. sp. *tritici* (*Pgt*) (44, 45). Virulent alleles for both genes were identified in *Pgt* races with diverse geographic origins. Whether these virulent alleles emerged before the introgression of *Sr35* and *Sr50* into wheat or as a consequence of their agricultural use was, however, not assessed. The identification of partially or fully virulent *AvrPm17* haplovariants (varB to varD) in a geographically diverse set of wheat mildew isolates collected over the last three decades and the identification of *AvrPm17* homologs in closely related mildew sublineages provided a unique opportunity to investigate a possible connection between the starting agricultural use of the *Pm17* introgression in wheat at the beginning of the 21st century and the emergence of virulent *AvrPm17* alleles in wheat mildew. Using 1) a global mildew population with a unique temporal resolution including many isolates that were collected before *Pm17* deployment or exhibit different host preferences, 2) careful phenotypic studies on transgenic *Pm17* lines, and 3) functional studies of *Avr* recognition in transient protein expression assays, we could demonstrate that virulent *AvrPm17* variants were largely present in mildew populations prior to the deployment of *Pm17*. We propose that this genetic diversity has arisen from the evolutionary arms race between *Blumeria* and its host species potentially tracing back to a *Pm17/Pm3*-like gene in the progenitor of rye and wheat. This hypothesis is corroborated by the fact that the *AvrPm17* gene is encoded in a highly expanded gene cluster of effector family E003, which is exclusive to wheat and rye mildew, suggesting that the expansion of this cluster evolved prior to the split of the two mildew lineages 250,000 y ago (23). One of the mechanisms that is proposed to drive expansion of effector gene clusters is the continuous coevolution with the host immune system (46, 47). Thus, the presence of *Pm17/Pm3*-like genes in the progenitor of rye and wheat might have resulted in selection pressure leading to the expansion of the effector cluster on chromosome 1 in the progenitor of wheat and rye mildew, suggesting a long history of *R* gene–mediated effector evolution in natural ecosystems, long before the start of agricultural cultivation. Our findings highlight the importance for resistance durability to select introgressed resistance specificities based on the evolutionary history of donor and recipient species. In this work, we demonstrate the need to identify and monitor the genetic diversity of the corresponding avirulence factors in order to achieve effective and durable resistance. We propose that such studies are very timely, considering the current important efforts to introgress *R* genes into wheat from phylogenetically distant wild relatives or phylogenetically close diploid progenitor species.

An often-stated advantage of larger translocations from related species is the simultaneous introgression of several resistance specificities active against different plant pathogens, such as the most widely deployed rye translocation 1BL.1RS from ‘Petkus’ carrying *Lr26*, *Yr9*, *Sr31*, and *Pm8* (4). For effective and durable resistance, the introgression of several resistance genes active against the same pathogen is highly desirable. By extending the mildew QTL mapping approach from *Pm17* transgenic lines to the original 1RS.1AL translocation cultivar ‘Amigo’, we have found evidence for the presence of a second resistance gene potentially recognizing an avirulence gene of *B.g. tritici* isolate Bgt_96224. Historically, the *Pm17* resistance associated with the 1RS.1AL translocation has been attributed to a single locus (48). The additional resistance specificity predicted by our QTL approach is therefore most likely genetically linked with the *Pm17* gene and has been missed by genetic approaches solely applied on the plant side due to suppressed recombination within the translocated genomic region originating from ‘Insave’ rye (37). The simultaneous presence of two race-specific resistance genes in the 1RS.1AL translocation might explain the initially broad resistance exhibited by cultivars such as ‘Amigo’, despite the likely long-standing presence of several gain-of-virulence alleles for *AvrPm17* in the *B.g. tritici* population. The identification of this second so far unknown AVR/R gene pair in the future will potentially provide further answers regarding the initial efficacy but also the quick breakdown of the powdery mildew resistance encoded on the 1RS.1AL translocation.

Identification and cloning of introgressed resistance genes has often been hampered by the absence of recombination throughout parts or the entirety of the alien chromatin regions (49). In recent years, several technological advances such as resistance gene enrichment sequencing or mutant chromosome sequencing (MutChromSeq) approaches that do not rely on fine mapping have helped to alleviate this phenomenon and led to the identification of numerous new resistance genes often residing in highly complex loci (50, 51). Here we show that genetic mapping populations of plant pathogens could provide an additional tool to dissect complex translocated genomic regions with low or absent recombination, thereby complementing recently developed, plant-focused approaches.

## Materials and Methods

Complete details are provided in *SI Appendix*, *Materials and Methods* (individual sections are cited here). Constructs used in this study are listed in Dataset S1. Primer sequences are listed in Dataset S2. Details about powdery mildew isolates and their associated (Sequence Read Archive) accession numbers are listed in Dataset S3. Phenotyping and subsequent QTL analysis are described in *SI Appendix*, section 1. Candidate identification is described in *SI Appendix*, section 2. Construction of the expression plasmids is described in *SI Appendix*, section 3. Transient expression procedures using *A. tumefaciens* in *N. benthamiana* followed by HR measurement are described in *SI Appendix*, section 4. Western blot detection of tagged avirulence and resistance genes can be found in *SI Appendix*, section 5. Expression analysis can be found in *SI Appendix*, section 6. PCR-based dissection of the *AvrPm17* locus can be found in *SI Appendix*, section 7. Bioinformatic analyses are detailed in *SI Appendix*, section 8. The wheat protoplast assay is described in *SI Appendix*, section 9. The sequence of *Pm17* is available in GenBank under accession number AYD60116.1. The AvrPm17 haplovariants are available in GenBank under accession numbers OM258717 to OM258731.

## Supplementary Material

Supplementary File

Supplementary File

Supplementary File

Supplementary File

## Data Availability

Genomic resequencing data for powdery mildew strains and PacBio sequencing data for *B.g. tritici* isolate ISR7 have been deposited in the Sequence Read Archive (accession nos. PRJNA625429 and PRJNA783175) (52, 53). The genome assembly of *B.g.* *tritici* isolate ISR7 was deposited in the European Nucleotide Archive (accession no. PRJEB41382) (54). The *Pm17* sequence is available in GenBank (accession no. AYD60116.1) (55). The AvrPm17 haplovariants were deposited in GenBank (OM258717–OM258731) (56, 57). Scripts and data used to produce the figures have been deposited to GitHub (https://github.com/MarionCMueller/AvrPm17) (58).
