# Supplementary material for: Ancient variation of the AvrPm17 gene in powdery mildew limits the effectiveness of the introgressed rye Pm17 resistance gene in wheat
Source: Proc Natl Acad Sci U S A. 2022 Jul 20;119(30):e2108808119. doi: 10.1073/pnas.2108808119 (PMC9335242; doi:10.1073/pnas.2108808119)
Supplement: Supplementary File [file pnas.2108808119.sapp.pdf]

Supplementary Information for

Ancient variation of the *AvrPm17* gene in powdery mildew limits the effectiveness of the introgressed rye *Pm17* resistance gene in wheat

Marion C. Mueller[#1]*, Lukas Kunz[#1], Seraina Schudel[1], Aaron W. Lawson[2], Sandrine Kammerecker[1], Jonatan Isaksson[1], Michele Wyler[1], Johannes Graf[1], Alexandros G. Sotiropoulos [1], Coraline R. Praz[1], Beatrice Manser[1], Thomas Wicker[1], Salim Bourras*[1, 3], Beat Keller*[1]

[1]Department of Plant and Microbial Biology, University of Zurich, Zurich, Switzerland
[2]Max Planck Institute for Plant Breeding Research, Cologne, Germany
[3]Department of Forest Mycology and Plant Pathology, Swedish University of Agricultural Sciences, Uppsala, Sweden

[#] Authors contributed equally. * Corresponding authors: Beat Keller, Salim Bourras, Marion C. Mueller

Email: bkeller@botinst.uzh.ch, salim.bourras@slu.se, marion.mueller@botinst.uzh.ch

This PDF file includes:

Supplementary Text 1-3
Material and Methods
Figures S1 to S22
Tables S1 to S7
Legends for Datasets S1 to S3

Other supplementary materials for this manuscript include the following:

Datasets S1 to S3

Supplementary Text

Supplementary Text 1: Recurrent events of gene conversion shaped the evolution of the *AvrPm17* locus

Gene duplication followed by sequence diversification has been suggested as a driving force to increase genetic diversity (1). The *Blumeria graminis* effector complement is characterized by high rates of gene duplication (2). In line with this finding, both isolates Bgt_96224 and THUN-12 contain an invertedly duplicated region of 2’300 bp of which 386 bp encode *AvrPm17* in a tail-to-tail orientation (Fig 3C). Interestingly, a similar orientation of identical effector genes is also found in four other candidate effectors pairs in in the genome of Bgt_96224 (Table S2). The high identity of the two copies of the coding sequences within the isolates (100% in Bgt_96224, 99% in THUN-12) indicated that the duplication is recent. However, when the analysis was extended to the entire duplicated region (813bp upstream and 1’100bp downstream both genes) we found that the sequences have considerably diverged (Fig 3D, Table S4, Fig. S16). The distal 200 bp segment of the 2’300 bp long duplication shares 79% and 84% identity upstream and downstream respectively (Table S4). The assembly of the newly sequenced isolate ISR7 also revealed two *AvrPm17* gene copies. In this isolate, the two *AvrPm17* gene copies are again oriented in the same tail-to-tail configuration as in Bgt_96224 and THUN-12. *AvrPm17*_ISR7 gene copies encode for identical proteins that differ by one amino acid (A53V) from AVRPM17_THUN-12 (Figure 3E). Most importantly, in all three genomes, each of the two duplicated regions carries a specific insertion that distinguishes the two duplicated segments (Fig. 3E, S15). In all three isolates, the downstream region of Copy1 of *AvrPm17* contains a 337 bp long insertion (termed insertion 1) that is absent downstream of Copy2. Vice-versa, insertion 2 (238 bp) is specifically present downstream of *AvrPm17_Copy2* in all three isolates but absent in *AvrPm17_Copy1*. Taken together, the high sequence divergence in the flanking regions and the presence of the duplicate-specific insertions indicates that the duplication is older than estimated based on *AvrPm17* gene identity and has existed in the ancestor of the three isolates. A mechanisms that best explains this structure is gene-conversion, a phenomenon that leads to non-reciprocal exchange of DNA between homologous sequences (3). Experimentally determined size of gene conversion (gene conversion tracts) in yeast are generally small (2kb) (4), however due to the small size of effector genes (<500bp), entire genes can be homogenized by gene conversion. In the case of *AvrPm17*, the data suggest the locus has undergone re-occurring gene conversion events between the two paralogous copies probably due to selection pressure to maintain the middle part of the duplication, containing the open reading frame, identical (Fig. 3D).

The genic SNP-pattern in the *AvrPm17* genes revealed by the three high quality genome sequences of *B.g. triticale* THUN-12, *B.g. tritici* Bgt_96224 and *B.g. tritici* ISR7, provides further evidence for gene-conversion events. Considering the avirulent THUN-12 as reference, both gene copies in Bgt_96224 have the same four SNPs (three in exons and one in the intron) (Fig. 3C,

S14). Similarly, both copies of *AvrPm17* in ISR7 carry two identical SNPs (Figure 3E). The duplications in both Bgt_96224,THUN-12 and ISR7 are however identical in size and orientation, indicating that the duplication must have existed in the ancestor of the three isolates. The most likely scenario is that the SNPs occurred in one gene copy and were then transferred to the second copy by independent gene conversion events, giving rise to the *AvrPm17* haplovariants varA/varA, varB/varB and varC/varC. In Bgt_96224, this possibly represents a relatively recent non-allelic gene-conversion event as the two gene copies as well as their 100 bp up- and 100 bp downstream region show complete sequence identity within the otherwise more diverged duplicated region (Fig. S14). Thus, we propose that non-allelic gene conversion played an important role in the *AvrPm17* evolution, by transferring beneficial mutations that occurred in one copy to its duplicate. Such a case has previously been described in *Phytophthora sojae*, where loss-of-recognition occurred in one copy of the tandemly duplicated *Avr3c* genes through amino acid substitution and was subsequently spread to the second gene copy via gene conversion (5). Gene duplications are considered advantageous for pathogens as they allow the independent accumulation of mutations and subsequent diversification of virulence factors (6). However, in case of recognition by a corresponding immune receptor the presence of identical avirulence gene copies represents a major disadvantage as gain of virulence mutations need to occur in both gene copies to escape recognition. This is exemplified by the tandemly duplicated *AvrPm3*^{d3} gene pair, in which one gene copy encodes amino acid polymorphisms leading to loss-of-recognition by *Pm3d* in numerous *B.g. tritici* isolates (7). However, the strains remain avirulent on *Pm3d* wheat lines due to the presence of a second avirulent *AvrPm3*^{d3} version. Thus, the spread of gain-of-virulence mutations from one gene copy to the second copy could represent a mechanism to cope with the adverse effect of encoding for multiple paralogous avirulence gene copies.

Supplementary Text 2: The ancestor of *B.g. tritici* encoded two *AvrPm17* copies

Using genomic mapping coverage as a proxy (2), we estimated that 91% of the globally collected 166 *B.g. tritici B.g. triticale* and *B.g. dicocci* isolates used in this study contain two *AvrPm17* genes. The only exceptions were the 11 isolates from China encoding *AvrPm17_varD* and an additional three isolates that likely encode more than two copies of *AvrPm17* (Fig. S20, SI appendix Dataset 3). Using genomic mappings, we were able to define the *AvrPm17* haplotype combination of 155 of these isolates (see SI appendix Dataset 3). Strikingly, 95% of the isolates with two *AvrPm17* copies encode for identical mature proteins in one of the following combinations; varA/varA, varB/varB and varC/varC (Fig. 4B). These findings indicate recurring gene-conversion events between *AvrPm17* paralogs and indicate the presence of a selection pressure, possibly linked to effector function in virulence, to maintain the sequences identical.

To determine if the isolates with one *AvrPm17* gene copy represent the ancestral state in wheat mildew, we compared the gene copies in Bgt_96224 and THUN-12 with isolate GZ-6 carrying the *varD* haplotype and *B.g dactylidis* that both contain a single *AvrPm17* gene. The 100bp sequence upstream of the start codon of *BgTH12-04537* contains nine SNPs compared to the corresponding region of *BgTH12-04538* and *Bgt-51729/Bgt-51731*; whereas the 100bp downstream are identical in all four genes (Fig S14). Interestingly, eight of the nine SNPs in the polymorphic 100bp upstream region of *BgTH12-04537* are found in the same region of *AvrPm17_Bgd* in *B.g. dactylidis* and of *AvrPm17_varD* in isolate GZ-6 (Fig S14). Minimal parsimony assumptions would suggest that isolates with a single *AvrPm17* gene represent the ancestral state prior to the duplication. However, alignment of the duplicated regions in Bgt_96224 and THUN-12 with the corresponding region in isolate GZ-6 (encoding varD) shows that the first 200 bp of the region in GZ-6 is identical to the first gene copy (*BgTH12-04537/Bgt-51729)* (100% and 99.5% identity, respectively, Table S4) whereas the 200 bp end of the segment is more similar to the second gene copy (*BgTH12-04538/Bgt-51731*) (90% identity compared to 86%) (Figure S16, Table S4). In addition, the downstream region of the gene in GZ-6 contains the same insertion (Insertion 2) as *BgTH12-04538/Bgt-51731* and lacks the insertion in *BgTH12-04537/Bgt-51729* (Insertion 1) (Fig. S15). This suggests that isolates containing a single *AvrPm17* gene represent a derived state that lost one copy possibly through recombination of the two paralogs. In summary, our data strongly suggest that the ancestor of *B.g. tritici* already encoded two *AvrPm17* paralogs that exchanged mutations through gene conversion.

Supplementary Text 3: QTL mapping of the *B.g. tritici* biparental F1 population derived from a cross of Bgt_96224 X THUN-12 on wheat cultivar 'Amigo'

We predicted the presence of an additional *R* gene in the original 1AL.1RS translocation line 'Amigo' which partially masks the effect of *Pm17* (8). We therefore phenotyped 117 progeny of Bgt_96224 X THUN-12 on cultivar 'Amigo' and observed highly quantitative segregation of the progeny (Fig. 5C). Subsequent single interval QTL mapping identified two significant QTLs, together explaining 75% of the phenotypic variance observed on cultivar 'Amigo' (Fig. 5D, Table S6). The first QTL is located on chromosome 9 at 340cM (LOD=15.97) whereas the second QTL locates to chromosome 1 at 165.8cM (LOD=4.24) which corresponds to the *AvrPm17* locus found on transgenic *Pm17* lines (Fig. 5D, Fig. 1A) thereby confirming the activity of *Pm17* in 'Amigo'. Since the highly significant QTL located on chromosome 9 was previously not found on *Pm17* transgenics, we hypothesized that it harbors an additional avirulence gene recognized by the predicted second resistance specificity active against *B.g. tritici* in the 1AL.1RS translocation of 'Amigo'. In this scenario the 'Amigo' avirulent isolate Bgt_96224 encodes for the avirulence component of this unknown resistance gene whereas THUN-12 encodes for the corresponding virulent allele. Indeed, segregation analysis of the progeny of Bgt_96224 X THUN-12 revealed that full virulence on 'Amigo' was specifically observed for progeny containing the *AvrPm17_96224* haplovariant and the THUN-12 genotype in the QTL on chromosome 9 (Fig. 5E, F).

The genetic confidence interval of the QTL on chromosome 9 (1.5LOD) delimited by markers snp106219 and snp106395 encompasses a physical interval of 371'034 bp in the *B.g. tritici* Bgt_96224 assembly (Table S7, Fig. S23A). The interval encodes 38 genes, of which 16 represent candidate effector genes encoding for 13 unique proteins (Fig. S23A, Table S7). Furthermore, two non-effector genes, *Bgt-3045* and *Bgt-3046*, contain a predicted signal peptide and are therefore likely secreted. The majority of the encoded candidate effectors in this locus (14 of 16) are part of candidate effector family E001, the largest candidate effector family in *Blumeria* (2). Interestingly, only five effectors are polymorphic between the isolates Bgt_96224 and THUN-12: *Bgt-51585*, Bgt-70025 and *BgtE-20010* contain one amino acid polymorphism in the mature peptide and *BgtA-21577* contains four. In addition, Bgt-70077 is duplicated in THUN-12 and the two proteins contain one and seven amino-acid polymorphisms compared to the 96224 protein, respectively (Table S7). To rule out the possibility that the QTL on chromosome 9 represents a second *Pm17* avirulence locus we co-expressed all 16 candidate effector genes as well as the presumably secreted *Bgt-3045* and *Bgt-3046* with *Pm17* in *N. benthamiana*. None of the candidate genes triggered a hypersensitive response (Fig. S23B). Thus, we conclude that the QTL on chromosome 9 does not represent an avirulence locus corresponding to the rye resistance gene *Pm17* but likely encodes an avirulence component recognized by a second *R* gene in the cultivar 'Amigo'.

In summary, we hypothesize that the original 1AL.1RS translocation present in cultivar ‘Amigo’ carries two *R* genes inherited from ‘Insave’ rye. This stands in contrast to previous reports which indicated a single powdery mildew resistance specificity (*Pm17*) in Amigo (9). However, the 1AL.1RS translocation has been described to display repressed rates of recombination (10) and to our knowledge, an analysis of individual mutants which would allow to dissect resistance specificities encoded by multiple linked *R* genes has never been performed on the 1AL.1RS translocation. Here we used a pathogen-based strategy using a segregating mildew population to decipher the two resistance specificities in ‘Amigo’.

SI appendix Material and methods

Section 1 Plant material, mildew isolates, phenotyping and QTL analysis

Crossing, genotyping and genetic map production of the mapping population Bgt_96224 X THUN-12 was described in (2). F1 progeny were phenotyped on the *Pm17*-donor line 'Amigo' and *Pm17* transgenic lines BW Pm17#34 and BW Pm17#181 described in (8) and scored at 10 days post infection as described in (7). The susceptible cultivar 'Kanzler' was used as infection control. Phenotypes were assessed for individual leaf segments according to the following scale: avirulent = 0, avirulent/intermediate 0.25, intermediate 0.5, intermediate/virulent =0.75, virulent = 1 and final score consist of an average of at least four leaf segments.

Single interval QTL mapping was performed using the R/qtl v.1.46.2 (https://rqtl.org/) package in Rstudio (v1.2.1335, (11). The genetic map was processed using the commands read.cross(), jittermap() and calc.genoprobe(, step=1). Due to the non-normalized distribution of the phenotypes, single interval analysis was performed using the scanone(model="np") method. For comparison to a parametric approach the sancone command was performed using the model="normal" option. Significance levels were established with 1000 permutations of the scanone command. Genetic confidence intervals were extracted using the command lodint(expandtomarkers=TRUE). Percentage of the variance explained by the two QTL was calculated using the command sim.geno(step=1, n.draws=100, err=0.01) followed by the command makeqtl(, chr=c(9,1), pos=c(340,165.7)) and fitqtl(method="hk",pheno.col=2, formula=y~Q1+Q2+Q1:Q2).

Natural *B.g. tritici* and *B.g. triticale* isolates were phenotyped on BW Pm17#181 and BW Pm17#34 and the corresponding sister lines as described above. Statistical significane of infection between the sister lines and *Pm17* transgenic lines was assessed in R studio (11) using the wilcox.exact() test included in the exactRankTests R package.

Section 2 Candidate identification

AvrPm17 candidate identification was based on the assembly and annotation of parental isolates Bgt_96224 (2) and THUN-12 (12). The physical intervals underlying the genetic confidence intervals were analysed manually, and correctness of the annotation was assessed using RNA-Seq data from both parental isolates (13, 14). For this purpose, RNAseq was mapped against the reference genome with STAR (v2.6.0a, (15)) using the method described in (16) and visualized with the integrative genome viewer IGV (v2.8.6, (17). Low quality gene models, overlapping with transposable elements, lacking transcriptional support or disrupted open-reading frames were excluded from the analysis. Erroneous gene models were corrected manually. Signal peptide prediction was performed with SignalP5.0 (18). Differential gene expression analysis was

performed following transcript quanitification with salmon (v1.3.0, (19) using default parameters using EdgeR (v3.11, (20)) as described in (16). LogFold change (logFC) values of >1.5 was considered as significant.

Section 3 Expression constructs

The molecular identification of the *Pm17* gene and C-terminal epitope tagging with a hemagglutinin (HA) epitope have been described in (8). *Pm17*-HA was transferred into a gateway compatible entry plasmid using the pENTR-D-TOPO Kit (Invitrogen) according to the manufacturer. Transfer of *Pm17*-HA into the binary expression vector pIPKb004 (21) was achieved using Gateway LR clonase II (Invitrogen). Expression constructs of epitope-tagged *Pm3* alleles and *Pm8* have been previously described in (7).

A complete list of all effector constructs produced by gene synthesis by our commercial partner BioCat (https://www.biocat.com) or site directed mutagenesis (SDM) can be found in SI appendix Dataset 1. All effector constructs were codon-optimized for *N. benthamiana* using the codon optimization tool of IDT (Integrated DNA technologies, https://eu.idtdna.com/) and synthesized with attL-sites. The constructs were cloned into the binary expression vector pIPKb004 (21) using LR clonase II (Invitrogen) according to the manufacturer and transformed into *A. tumefaciens* using the freeze-thaw protocol described in (22). PCR based SDM and epitope tagging was performed with non-overlapping primers on templates, both listed in SI appendix Dataset 2. Phosphorylation of the linear PCR product was performed with T4 polynucleotide kinase (New England Biolabs) and subsequently ligated with T4 DNA Ligase (New England Biolabs) according to the manufacturer.

Section 4 *Agrobacterium tumefaciens* mediated transient expression in *Nicotiana benthamiana*

Agrobacterium tumefaciens mediated transient co-expression of effector and resistance genes was conducted according to the protocol described in (7). To test for recognition, effector candidates and resistance genes were infiltrated at a ratio of R:effector 1:4 and incubated for 5 days followed by hypersensitive response imaging by the Fusion Imager FX system as described in (7). Effector genes that did not induce HR response under this standard condition were considered as non-recognised. To allow a quantitative comparison of the recognition strength between *AvrPm17* variants, HR induction was measured in pairwise tests using an infiltration ratio of *Pm17*: *AvrPm17* of 1:1 and infiltrated leaves were imaged at 2dpi to 5dpi depending on the strength of hypersensitive response reaction. HR was subsequently quantified using Fiji (23)) according to the procedure described in (7). Statistical significance was assessed performing a paired wilcoxon rank sum test in Rstudio (v1.2.1335, (11) using the command wilcox.test(paired = TRUE).

Section 5 Western blot analysis

Protein extractions were performed according to (7) from eight pooled leaf discs (5mm diameter) originating from four *Agrobacterium* infiltrated leaves at 2dpi. Leaf discs were ground in 150µl 2XLaemmli buffer (100 mM Tris-HCl pH 6.8, 200 mM DTT, 0.04% bromophenol blue, 20% glycerol, 2% SDS), heated to 95°C for 5min, followed by a centrifugation of 10min at 10’000xg. SDS polyacrylamide (PA) gels were used to separate 10 µl of total protein extract (8% PA for PM17-HA and 16% for AVRPM17-FLAG variants), followed by semi-dry blotting on a nitrocellulose membrane (Amersham Protran 0.2 µm NC) using the Trans-Blot SD Semi-Dry Transfer Cell from Bio-Rad. To control for equal loading, the membrane was stained by Ponceau-S. Subsequent detection of HA-tagged proteins was done with a peroxidase-conjugated antibody (anti-HA-HRP, rat monoclonal, clone 3F10, Roche) at a dilution of 1:3000. The FLAG epitope tag was detected with the primary antibody (anti-FLAG, mouse monoclonal, clone M2, Sigma-Aldrich, 1:10’000 dilution). After washing the membrane with TBS-T, the membrane was incubated with anti-mouse peroxidase antibody (anti-mouse-HRP, goat polyclonal, Sigma Aldrich) at a dilution of 1:4000. For chemiluminescence detection, we used the WesternBright ECL HRP substrate (Advansta) and detected the signal with Fusion FX Imaging System (Vilber Lourmat, Eberhardzell, Germany).

Section 6 Expression analysis

To verify expression of *AvrPm17* in a selected subset of 16 *B.g tritici* and *B.g. triticale* isolates (Fig. S19) RNA was extracted from leaf segments of the susceptible wheat cultivar ‘Chinese Spring’ 48h after infection with the indicated powdery mildew isolate using the SV total RNA Isolation System Kit (Promega) according to the manufacturer. Uninfected ‘Chinese Spring’ was used as a negative control. Total RNA purity and integrity was verified using a Nanodrop1000 (Thermo Scientific) and agarose gel electrophoresis respectively. Total RNA was reverse transcribed using iScript Advanced cDNA Synthesis Kit for qRT-PCR (Promega) according to the manufacturer. As a negative control, the same reaction was performed in the absence of the reverse transcriptase (‘RTminus’). *AvrPm17*, *AvrPm2* and fungal *GAPDH* were amplified from cDNA using Phusion HF DNA Polymerase (New England Biolabs) and the primers listed in SI appendix Dataset 2. The same reactions were performed on the ‘RTminus’ samples resulting in no detectable PCR product thereby ruling out any contaminations with genomic DNA.

As control, we also performed PCR amplification of *AvrPm17*, *AvrPm2* and fungal *GAPDH* from genomic DNA using the same experimental procedure as described for the cDNA samples above. For extraction of the genomic DNA the susceptible wheat cultivar ‘Chinese Spring’ was infected with the *B.g. tritici* isolate 96224 or the *B.g. triticale* isolate THUN-12 as described above. DNA was extracted 48h after infection using a guanidine-thiocyanate based protocol previously described in (24). Uninfected ‘Chinese Spring’ was used as a negative control. Total genomic DNA purity and

integrity was verified using a Nanodrop1000 (Thermo Scientific) and agarose gel electrophoresis respectively. PCR amplification of *AvrPm17*, *AvrPm2* and fungal *GAPDH* from genomic DNA was performed as described for the cDNA samples above.

Section 7 PCR based dissection of the *AvrPm17* locus

For extraction of genomic DNA the susceptible cultivar ‘Bobwhite’ was infected with the indicated *B.g. tritici* or *B.g. triticale* isolates as described above. DNA was extracted 48h after infection using a guanidine-thiocyanate based protocol previously described in (25). Uninfected ‘Bobwhite’ was used as a negative control. PCR amplifications specific for different regions of the complex *AvrPm17* locus were conducted with the primers indicated in Fig. S15 and Dataset S2 using Phusion High-Fidelity DNA Polymerase (New England Biolabs) according to the manufacturers protocol and visualized on a 1% agarose gel. Subsequently, all obtained PCR amplicons were subjected to Sanger sequencing in order to verify *AvrPm17* coding sequences, as well as presence and exact position of the copy-specific insertions to match the respective reference assemblies of Bgt_THUN-12, Bgt_96224, Bgt_ISR7 or Bgt_GZ-6 representing the four *AvrPm17* haplovariants varA-varD.

Section 8 Bioinformatics analysis

Genomic origin of the *AvrPm17* locus

Genomic origin of the *AvrPm17* locus was assessed according to fixed polymorphism in *B.graminis formae specialis* (f.sp*)* following to the rationale described in (14): SNP call was performed on isolates using freebayes (v1.1.0-54-g49413aa, (26)) with the -ploidy 1 setting. Isolates used for this analysis are indicated in SI appendix Dataset 2. The SNP dataset was filtered with vcftools (v0.1.5, (27)). Only SNPs with sequencing depth 20 and quality above 20 were used. Polymorphisms were considered fixed if they were present in all isolates of one f.sp. and absent in all the other ff.spp*.* The polymorphisms found in THUN-12 were compared to this set of fixed polymorphisms. Genomic coverage was calculated using SamTools depth command –a per position and normalized to the average coverage of chromosome 01. Normalized genomic coverage was visualized in 1000bp windows.

Structural modelling

Secondary structure prediction was performed using the QUICK2D toolkit (28). Tertiary protein structure modelling was performed using the IntFOLD5.0 server (https://www.reading.ac.uk/bioinf/IntFOLD) (29)

Phylogenetic tree

Candidate effectors of the family E003 in *B.g. tritici* 96224 and *B.g. triticale* were identified as described in (2). To identify E003 family members in *B.g. hordei* DH14, the proteins of DH14 were aligned to the E003 effectors using BLASTP+ (v2.6.0, (30)) and hits e-value greater than e^{-10} were retained. Amino acid sequences of the effectors were aligned with MUSCLE (v3.8.31, (31)). The approximate maximum-likelihood of the phylogenetic relationships were subsequently investigated with the Jones-Taylor-Thorton algorithm implemented with FastTree (v2.1.8, (32)). The local support values of the nearest-neighbor interchanges topology were calculated with the Shimodaira-Hasegawa test from FastTree (v2.1.8,(33). Rooting with the outgroup BGTE-20002 and the graphical representation of the tree were accomplished with FigTree (v1.4.3, http://tree.bio.ed.ac.uk/software/figtree).

Cluster analysis

To identify syntenic scaffolds between *B.g. tritici/B.g. triticale* and the *B.g.hordei* assembly DH14, we used the Orthofinder tool (v2.3.3, (34)) to identify single orthologous genes in the proteome of *B.g. tritici* 96224 (2) , *B.g. triticale* THUN-12 (12) and *B.g. hordei* DH14 (35) and *B.g. hordei* RACE1 (2, 35). A scaffold in DH14 was assigned to a chromosome of *B.g. tritici/B.g. triticale* if the classification was supported by the majority of the single orthologous genes on the scaffold. Subsequent detailed synteny analysis in the *AvrPm17*-locus was done manually using the

integrated genome viewer (IGV, v2.8.6, (17) and genes were considered as syntenic based on conserved orientation and placement in the same orthogroup by OrthoFinder. Low-quality gene models were removed from the analysis if they overlapped with TE, showed no transcriptional support or lacked a start/stop codon. To predict presence/absence of the genes in *B.g. secalis*, Illumina sequences were mapped against the Bgt_genome_v3_16 as described in (2) and copy number variation was estimated using the genomic coverage method described in (2). Therefore, the average number of reads mapping to the genic region of all genes (including intron) were calculated using the SamTools depth –a (v1.7,(36)) command. Average coverage was normalized to the average coverage of all genes. Genes with normalized coverage <0.1 were considered absent. Genes were considered present in the *B.g. secalis* f.sp. if the gene was present in at least one isolate of *B.g. secalis*.

Orientation of candidate effector pairs

Identical protein sequences of 844 Bgt_96224 candidate effectors (Müller et al., 2019) were identified using blastp (v2.6.0 (30) using the qcov_hsp_perc 100 option. Hits were subsequently filtered for hits with a identity of 100%. Gene position was manually assessed and orientation of gene pairs was determined as follows; relative gene orientation was determined if two candidate effectors were located right next to each other or only separated by short low-confidence genes.

Haplotype analysis

Haplotype analysis was conducted based on the re-sequencing data of 166 *B. graminis* isolates as listed in SI appendix Dataset 3. Sequencing of the isolates that were published prior to this study are described in (7, 13, 14). DNA for isolates that were sequenced for this study was extracted using a chloroform/phenol method described in (37) and sequencing was performed as PE150 on the Illumina HiSeq4000 at the functional genomic center Zurich (FCGZ). For the haplotype analysis in the worldwide *B. graminis* population, genomic re-sequencing data was mapped against the Bgt_genome_v3_16 as with bowtie2 (v2.2.9.(38) using the parameters in (2). Illumina sequences with bad read quality were processed with trimmomatic (v0.38, (39)) with LEADING:3 TRAILING:3 SLIDINGWINDOW:4:20 MINLEN:50 commands prior to mapping as indicated in SI appendix Dataset 3. Mappings were subsequently processed with SamTools (v1.6 (36) view, sort and rmdup command as well as with the picard (2.16.0, (http://broadinstitute.github.io/picard) AddOrReplaceReadGroups command. Copy number of *AvrPm17* genes was estimated using the coverage-based method described in (2) Isolates with a normalized *AvrPm17* coverage of 1.6-2.4 were considered to contain two *AvrPm17* copies (also verified by PCR see Methods on PCR-based dissection of the *AvrPm17* locus above) Isolates with normalized coverage of 0.6-1 were

considered to contain a single *AvrPm17* copy. To exclude strain admixture, the rate of heterozygous SNPs in single copy orthologous genes shared in *B.g. tritici* and *B.g. hordei* was performed as follows: OrthoFinder (v2.3.3, (40) was used to extract single copy genes between Bgt_96224 (2) and DH14 (41) proteins. SNP call was performed in regions between the start and stop codon of the 5270 single copy orthologous genes with SamTools mpileup (v1.7 (36)) followed by the bcftools -mv command. A SNP was considered heterozygous if the minor allele was supported by more than 25% of the reads. Isolates with more than 5% heterozygous SNPs were excluded from the haplotype analysis.

SNP pattern in the genes *Bgt-51729/Bgt-51731* was assessed manually upon visualization in integrative genome viewer IGV (v2.8.6, (17). For isolates where SNP patterns indicate the presence of two distinct *AvrPm17* copies, re-sequencing data were mapped against a single *AvrPm17* gene copy (including 1000bp up and downstream of the gene) and analysed manually with the same approach as described above. Only isolates for which haplovariants could be assigned unambiguously were retained in the analysis. Haplovariant from *B.g. dactylidis* were extracted by blasting against a previously published de-novo assembly with BLASTN+ (v2.6.0,(30)).

Gene conversion

The duplicated regions in the genomes of Bgt_96224 and THUN-12 were aligned using dotter (v4.44.1(42)). The boundaries of the duplicated region were defined manually based on the analysis of the pairwise dot plots, as well as the identification of gene specific insertions. To enable pairwise alignment of the sequences, the insertions identified in Fig. S15 and Fig. S21 were removed. Subsequent pairwise alignment was performed with MUSCLE and nucleotide identity in 50bp sliding windows were calculated using a custom R script. To compare the *AvrPm17* gene of the high quality assemblies of Bgt_96224 and THUN-12 with isolates that carry one gene copy, we performed de-novo assemblies of isolate GZ-6, (see SI appendix Dataset 3) with *SPAdes* (v3.12.0, (43) with the default parameters. BLASTN+ (v2.6.0., (30)) was used to identify the contig containing the *AvrPm17* gene.

ISR7 genome assembly

DNA of fungal spores of isolate ISR7 were extracted as described in (2). Genome sequencing was performed on three SMRT cells using the PacBio Sequel technology at the Functional Genomic Center Zürich, Switzerland (FGCZ). Raw reads are available at the SRA (accession number: PRJNA783175). Genome assembly was performed with HGAP4 (SMRT Link Version 7.0.1.66975) with genome size 165000000 option. Detailed parameters can be found at: https://github.com/MarionCMueller/Bgt_ISR7_genome. The ISR7 assembly is available at the European Nucleotide Archive under accession number: PRJEB41382. The duplicated segments

containing *AvrPm17* genes were identified using BLASTN+ (v2.6.0., (30)). Alignment of duplicated segments and annotation of insertions were performed in the CLC workbench 8.0 (QIAGEN).

Presence of *Pm17* in rye

To check for the presence of the *Pm17* gene in previously published rye genomes, we used the blastp (v2.6.0 (30) to blast the PM17 protein sequence (GenBank: AYD60116.1) against the assemblies of Lo7 (44) and Weining rye (45). To detect the presence of *Pm17* in the ten re-sequenced rye lines (published in (46)), the whole-genome sequencing data was mapped against the genomic sequence of *Pm17* (GeneBank ;MH077963.1) using the bwa mem command (47) with default parameters. Samfiles Reads with exact match to the *Pm17* genomic sequence were extracted from the sam files using the commands grep NM:i:0. Subsequently, the sam files were transformed to bam format using SamTools (v1.6, (36)view. BamTools (2.5.1, https://github.com/pezmaster31/bamtools) command bamtofastq was used to transform mapped reads into fastq format. At this stage, only mapped reads with both pairs aligning to the *Pm17* genomic regions were retained. De-novo assembly was performed on these reads using SPAdes (v3.12.0 with default parameters, (43). The *Pm17* gene was considered present in a rye line if the full length coding-sequence without any SNPs could be recovered from the resulting de-novo assemblies.

Section 9 Wheat protoplast assays

For the wheat protoplast assay, Pm17-HA and AvrPm17 constructs were cloned into the binary expression vector pIPKb002 (21) using LR clonase II (Invitrogen) according to the manufacturer. Control constructs (Sr35/AvrSr35) are described in (48). Wheat protoplasts were extracted from seedlings of wheat cultivar “Chinese Spring” as described in (49). Plasmid extraction and subsequent co-transfection and luciferase measurements were performed as described in (49) The following plasmid concentrations were used: Sr35/Pm17 = 2µg, AVRs = 5µg and pZmUBQ:LUC = 4µg.

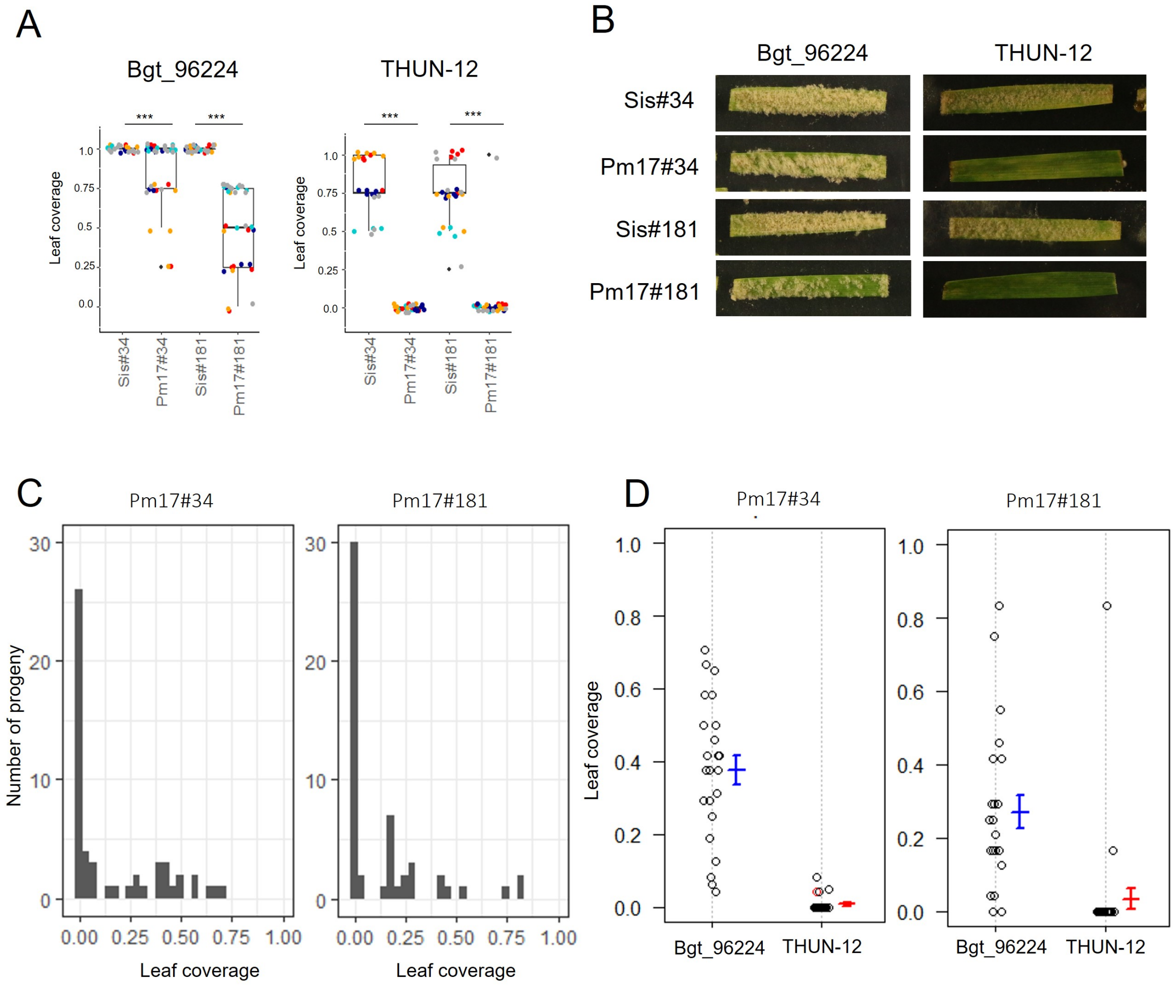

Fig. S1. Phenotypes of the mapping population Bgt_96224 X THUN-12 on two independent transgenic wheat lines expressing Pm17-HA (A) Phenotype of the parental isolates Bgt_96224 and THUN-12 on transgenic lines Pm17#34 and Pm17#181. Leaf coverage of individual leaf segments was scored according to the following scale: avirulent = 0, avirulent/intermediate 0.25, intermediate 0.5, intermediate/virulent =0.75, virulent = 1. Data from five independent phenotyping experiments are shown with at least 3 leaf segment per experiment scored. Significance was assessed using exact Wilcoxon rank test and significance levels are indicated above the boxplots as follows: *** p<0.001.(B) representative photographs of phenotypes of Bgt_96224 and THUN-12 on the two independent *Pm17* expressing transgenic lines and their corresponding sister lines at 10dpi. Data shown in Fig. S1A and S1B are also included as part of Fig. 4D for comparison. (C) Distribution of phenotypes of the 55 randomly selected progeny of the cross Bgt_96224 X THUN-12 after 10dpi. (D) phenotypes of the 55 progeny of the cross Bgt_96224 X THUN-12 that carry either the Bgt_96224 genotype or the THUN-12 genotype at the best associated marker of the QTL.

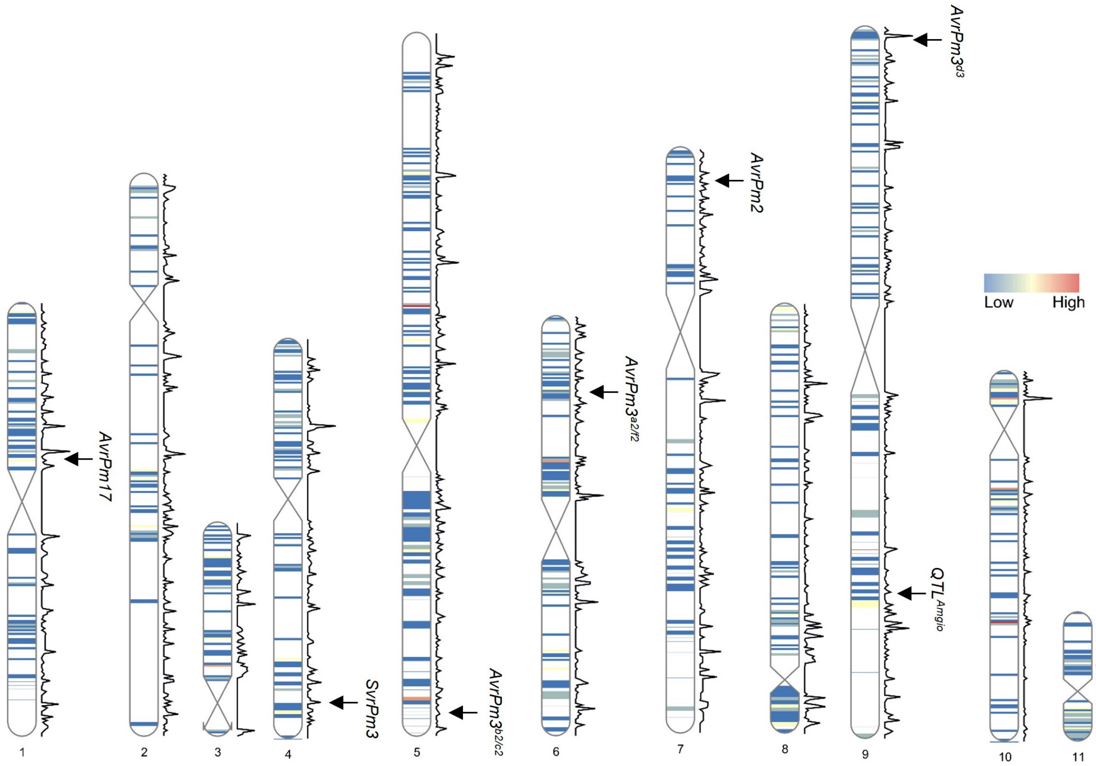

Fig. S2. Chromosomal location of *B.g. tritici* avirulence genes and the suppressor of avirulence *SvrPm3* in the assembly of isolate Bgt_96224. Chromosomes are colored according to effector gene density in 50kb windows. White areas represents intervals without effectors. Black line represents recombination rate in cM/50kb.

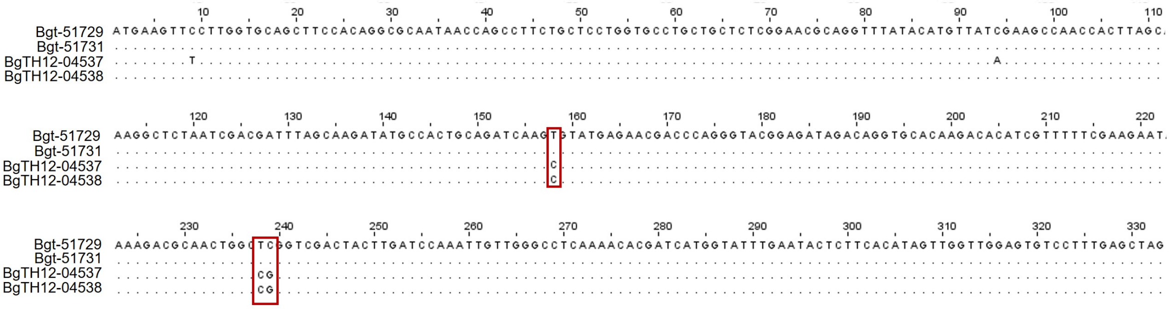

Fig. S3. Alignment of the nucleotide sequences of the two genes encoding for AVRPM17 in the isolates Bgt_96224 and THUN-12. Nucleotide differences that lead to amino acid changes are framed with a red box. BgTH12-05637/BgTH12-05438 and Bgt-51729/Bgt-51731 encode for identical proteins.

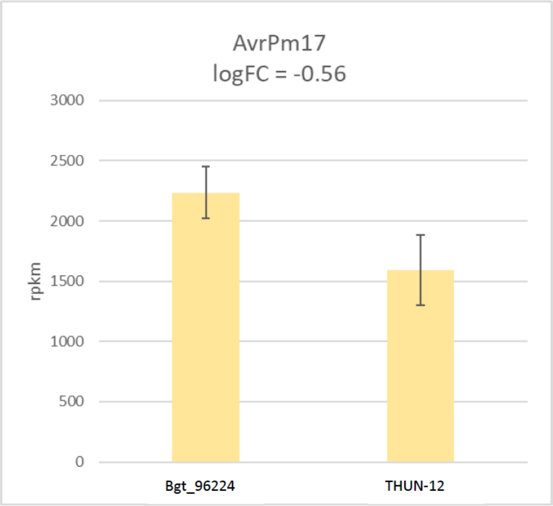

Fig. S4. Expression level of *AvrPm17* genes in the two parental isolates Bgt_96224 and THUN-12 using RNAseq data at 2 days post infection. Since *AvrPm17* is encoded by identical gene copies in the isolate Bgt_96224 and almost identical copies in THUN-12, expression levels can only be quantified combined for both gene copies. Expression levels are indicated as rpkm (reads per kilobase per million reads), logFC (log-fold changes) of >1.5 are considered significant.

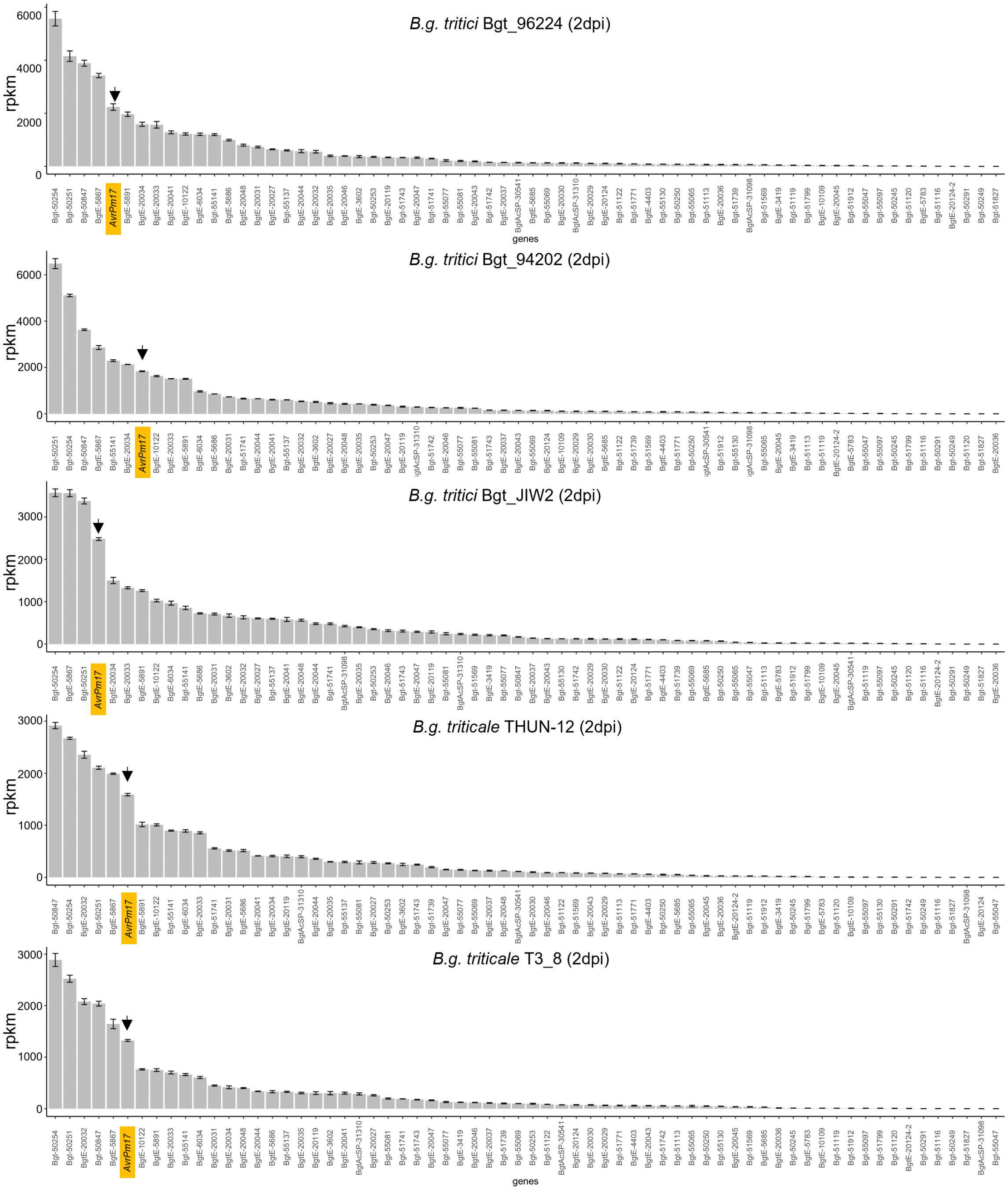

Fig. S5*.* Expression of genes encoding members of the candidate effector family E003 in three *B.g. tritici* isolates and two *B.g. triticale* isolates at 2dpi (days post infection) using RNAseq data. Expression levels are indicated as rpkm (reads per kilobase per million reads) and represent the average of three biological replicates for each isolate. *AvrPm17* (*Bgt-51729/Bgt-51731*) is highlighted in yellow.

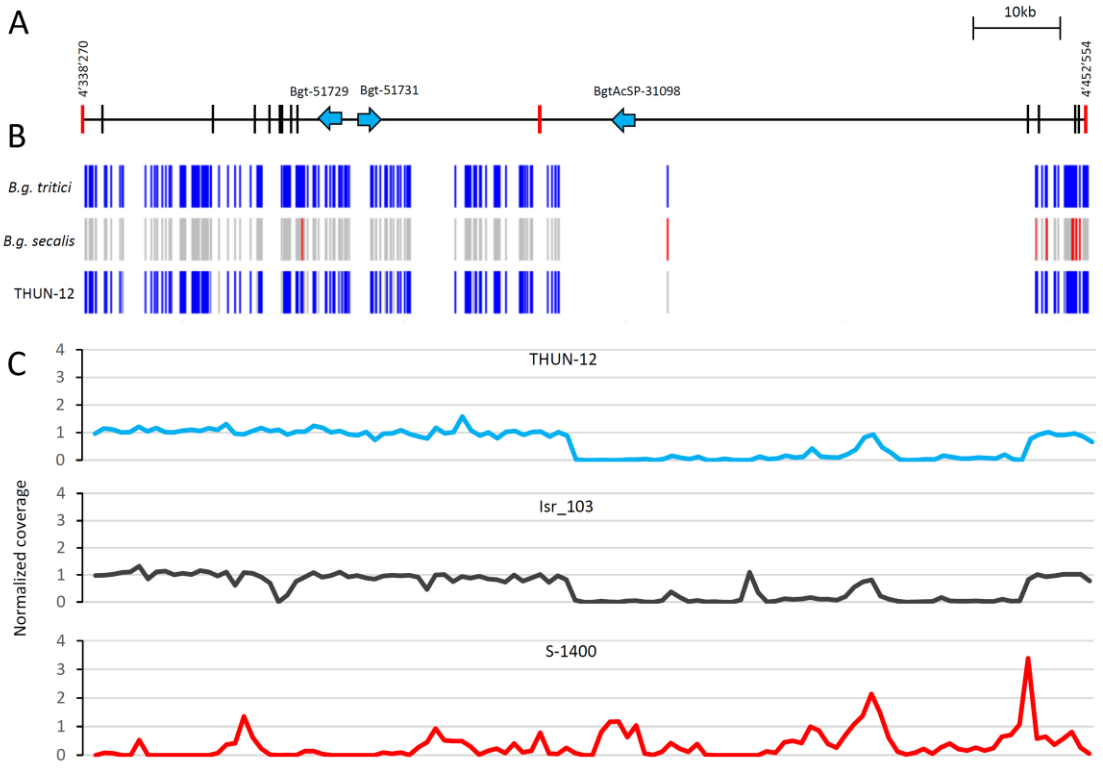

Fig. S6. Genomic origin of the *AvrPm17* locus on chromosome 01 in *B.g. triticale* isolate THUN-12. (A) representation of the physical interval of the *AvrPm17* locus in the Bgt_96224 genome assembly. Gene size is not drawn to scale. Bars represent informative markers in the genetic confidence interval (1.5LOD). Flanking markers and the best associated marker of the QTL are depicted in red. (B) Determination of genomic origin based on fixed polymorphisms within *formae speciales*. Blue bars represent polymorphisms fixed in *B.g. tritici* that differ from *B.g. secalis*, red lines represent polymorphisms that are fixed in *B.g. secalis* and differ from *B.g. tritici.* Grey indicates SNPs that could not be determined in *B.g. secalis* or THUN-12 because of lacking genomic coverage likely due to deletions of these particular regions. (C) Genomic coverage of the selected isolates *B.g. triticale* THUN-12, *B.g. tritici* Isr_103 and *B.g. secalis* S-1400. The 50kb deletion identified in *B.g. triticale* isolate THUN-12 can also be found in certain *B.g. tritici* isolates such as Isr_103. Normalized genomic coverage was estimated in 1000bp windows normalized to the average coverage of Bgt_chr-01.

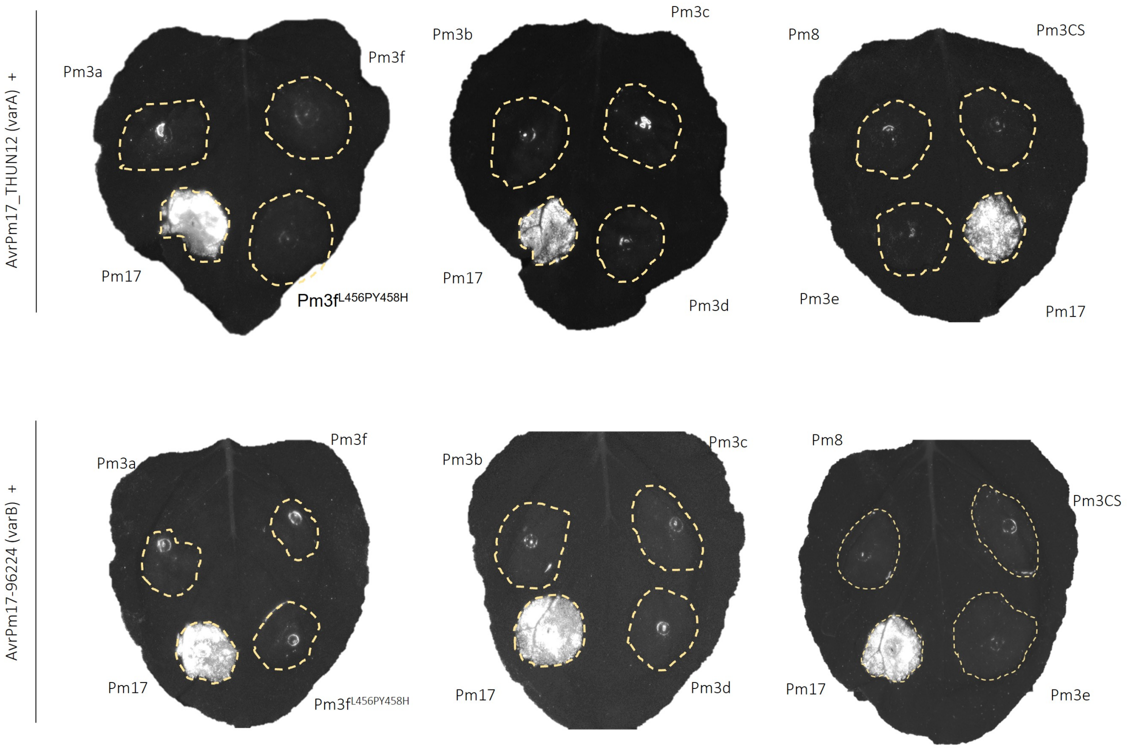

Fig. S7. AVRPM17 is not recognized by other members of the Pm3-NLR family. *AvrPm17_THUN12* (varA) and *AvrPm17_96224* (varB) were co-expressed with different alleles of the *Pm3/8/17* family. All infiltrations were done with the ratio R: Avr of 1:4 and imaged after 5dpi. Each infiltration was done with at least n=4 leaves and repeated twice.

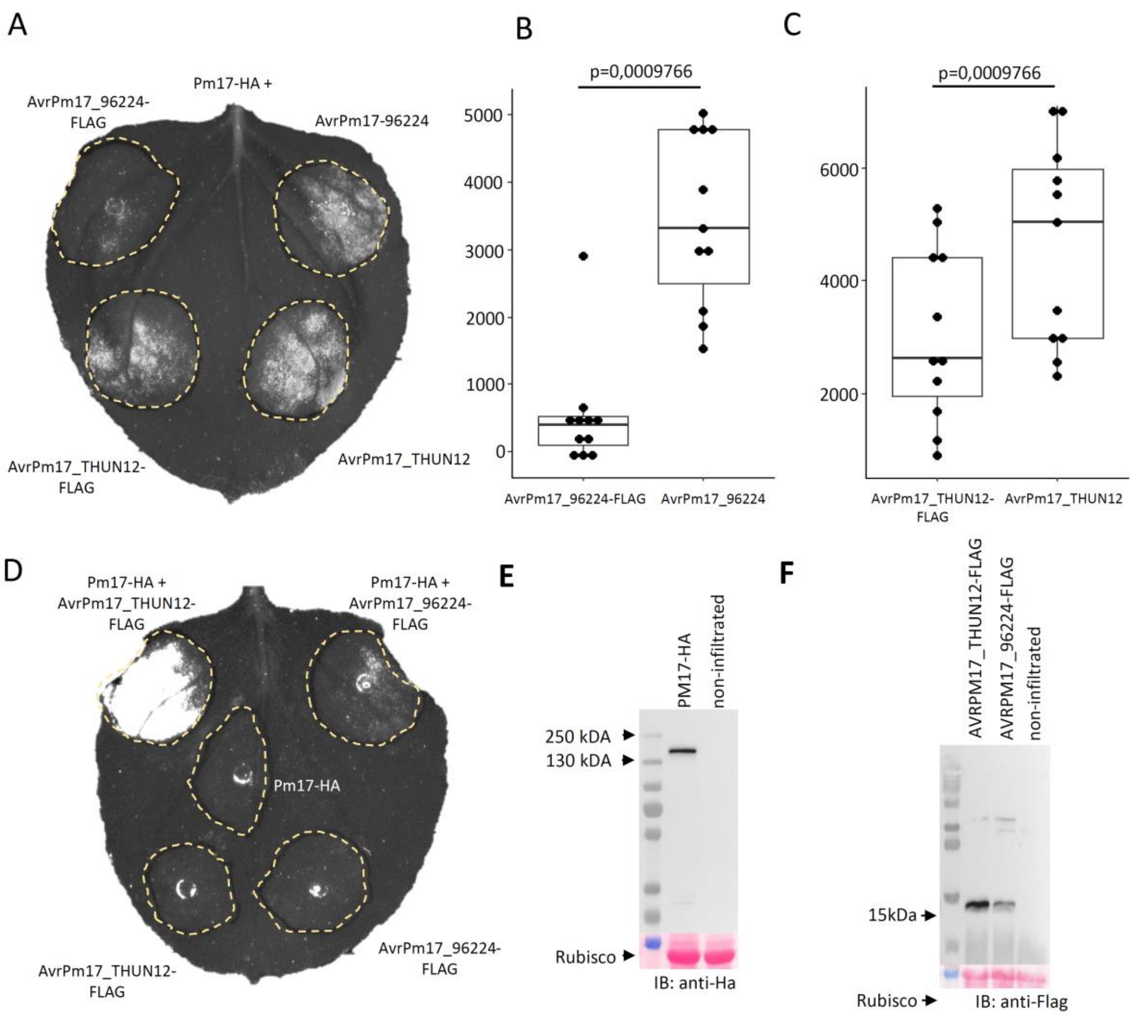

Fig. S8. Epitope tagging and western blot analysis of PM17 and AVRPM17 variants. (A-C) C-terminal FLAG-tag interferes with HR induction of AVRPM17 upon co-infiltration with PM17-HA. (A) shows a representative agrobacterium-infiltrated *N. benthamiana* leaf expressing Pm17-HA with FLAG epitope tagged and untagged versions. Pm17-HA and AVRPM17 variants were infiltrated at 1:4 ratios. (B,C) HR of n=10 leaves was quantified at 2dpi in two independent experiments. Statistical significance was accessed using the paired Wilcoxon ranked sum test, p-values are reported above the boxplots. (D) shows a representative picture of a agrobacterium-infiltrated leaf expressing PM17-HA with AVRPM17-FLAG variants at a R:AVR ratio of 1:6 and individual infiltrated constructs. HR of n=6 leaves was measured after 5dpi with the Fusion ImagerX system in two different experiments (E,F) western blot of Pm17-HA and AVRPM17-FLAG variants.

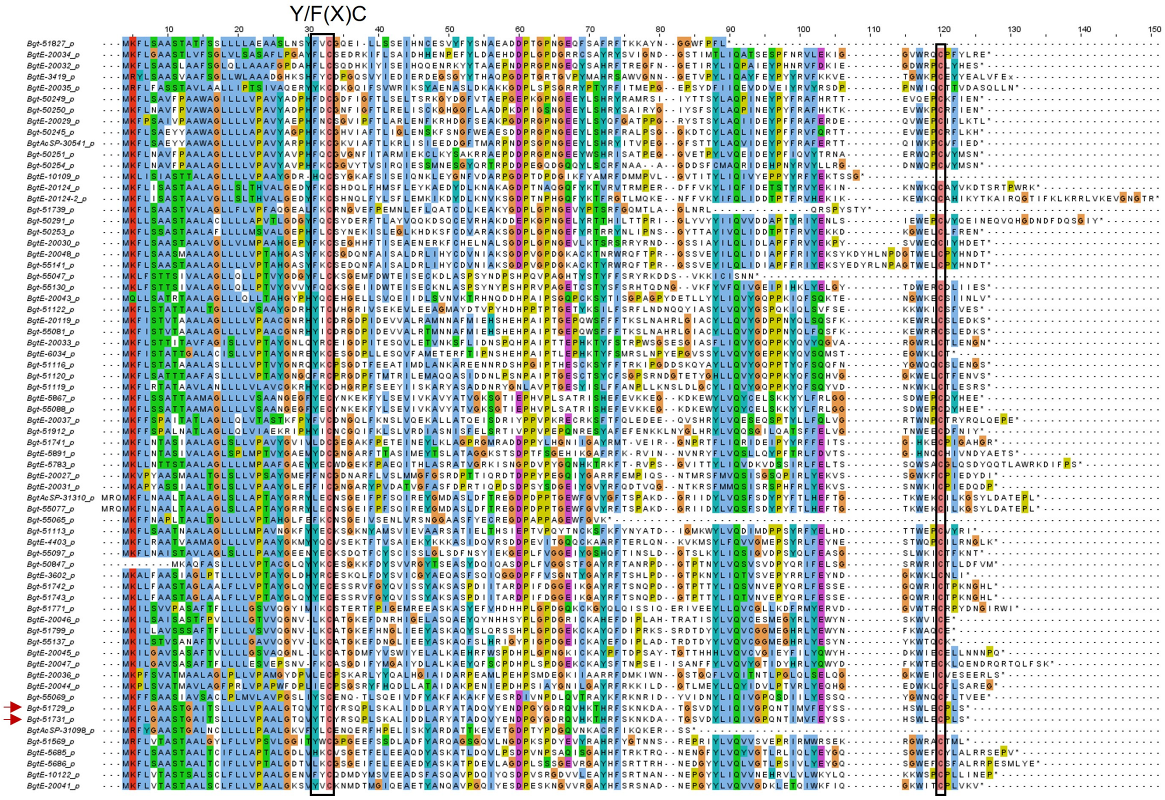

Fig. S9. Protein sequence alignments of the effector family E003 members found in the reference isolate Bgt_96224. AVRPM17 is highlighted by red arrows. The conserved Y/F(x)C motif and C-terminal cysteine are indicated (black boxes). The alignment was colored according to the ClustalX color scheme as follows: blue: hydrophobic; red: positively charged; purple: negatively charged; green: polar uncharged; yellow: proline; fleshy pink: cysteine; orange: glycine.

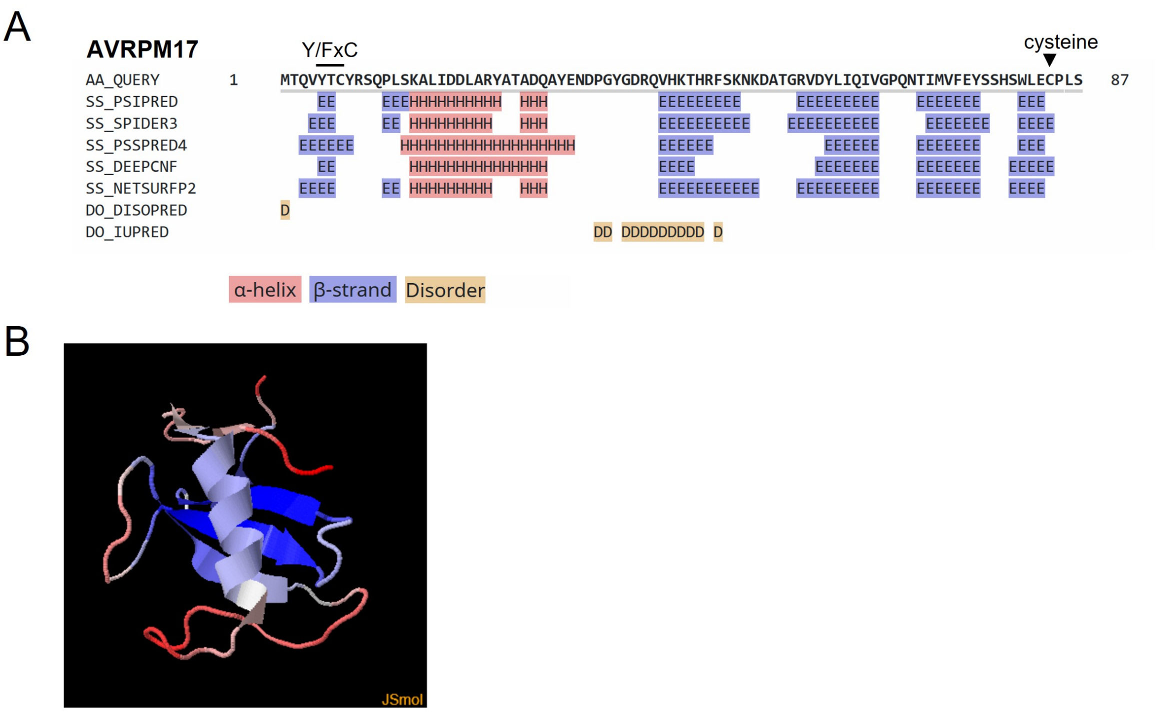

Fig. S10. Structural modelling of the AVRPM17 protein. (A) Modelling of the 2D protein structure of AVRPM17_THUN12 without signal peptide using the the Quick2D toolkit**.** Y/FxC motif and C-terminal cysteine are indicated (B) Representation of the best model (p value=1.145E-4) obtained by modelling the mature peptide of AVRPM17_THUN-12 with IntFOLD5.0. The model is based on the following templates: fungal RNAse po1 from *Pleurotus ostreatus* (3whoA, described in (50)) and BEC1054 an effector from *B.g. hordei* (6fmbA, described in (51)) The model is colored according to the JSmol coloring scheme in which blue is designating high model accuracy and red low model accuracy.

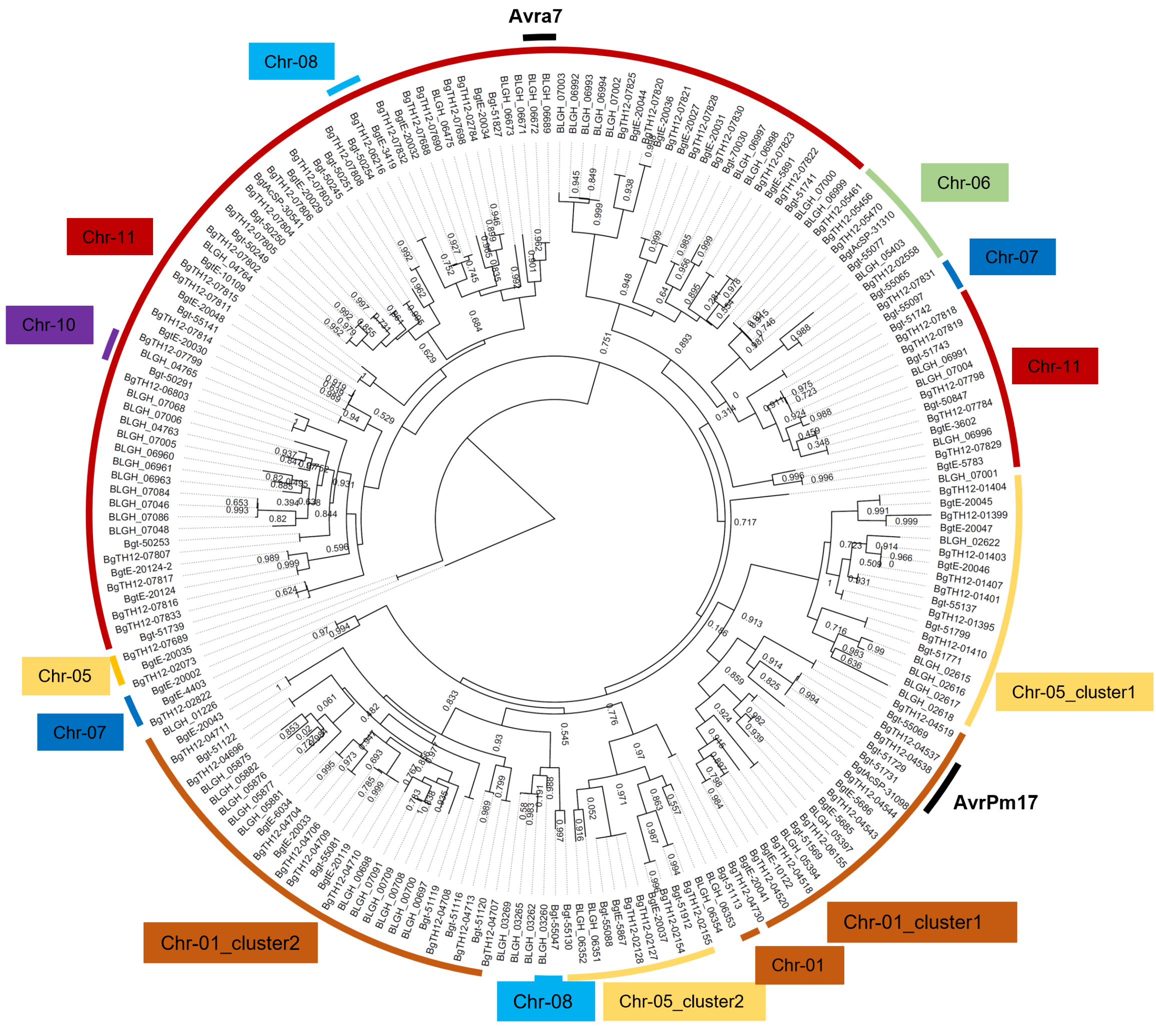

Fig.S11. Phylogenetic tree of the E003 family with 69 members in *B.g. tritici*, 70 members in *B.g. triticale* THUN-12 and 59 members in DH14. Gene clades were colored according to their position on the eleven chromosomes of *B.g tritici* and *B.g. triticale*. The local support values to the nearest-neighbor interchanges topology were calculated with the Shimodaira-Hasegawa test and indicated for each branch.

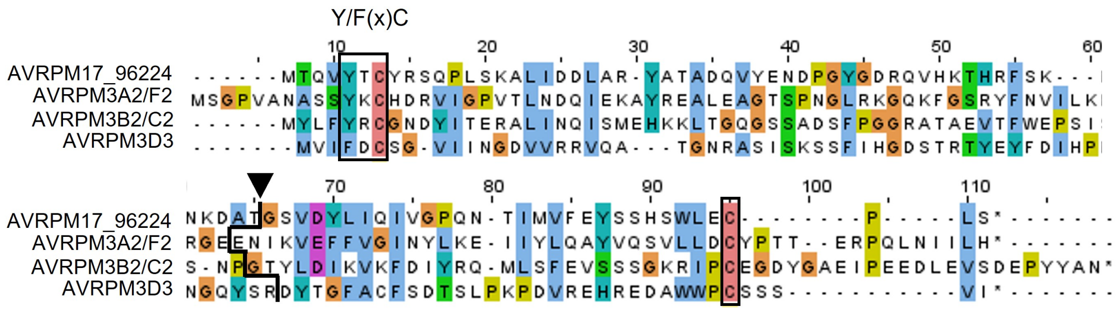

Fig. S12. Protein alignment of the mature protein (without signal peptide) of AVRPM17_96224, AVRPM3[A2/F2], AVRPM3[B2/C2] and AVRPM3[D3]. The Y/F(x)C motif and the conserved cysteine are indicated with black boxes. The intron position in AVRPM17 is indicated by a black arrow and by a black line in the AVRPM3s. The alignment was colored according to the ClustalX color scheme as follows: blue: hydrophobic; red: positively charged; purple: negatively charged; green: polar uncharged; yellow: proline; pink: cysteine; orange: glycine.

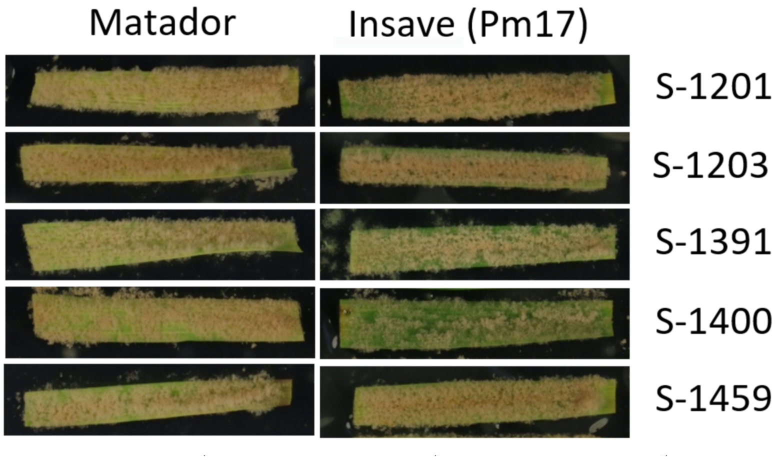

Fig. S13. Phenotypes of five *B.g. secalis* isolate on the rye *Pm17* donor ‘Insave’ and the susceptible rye cultivar Matador. Images were taken at 10dpi.

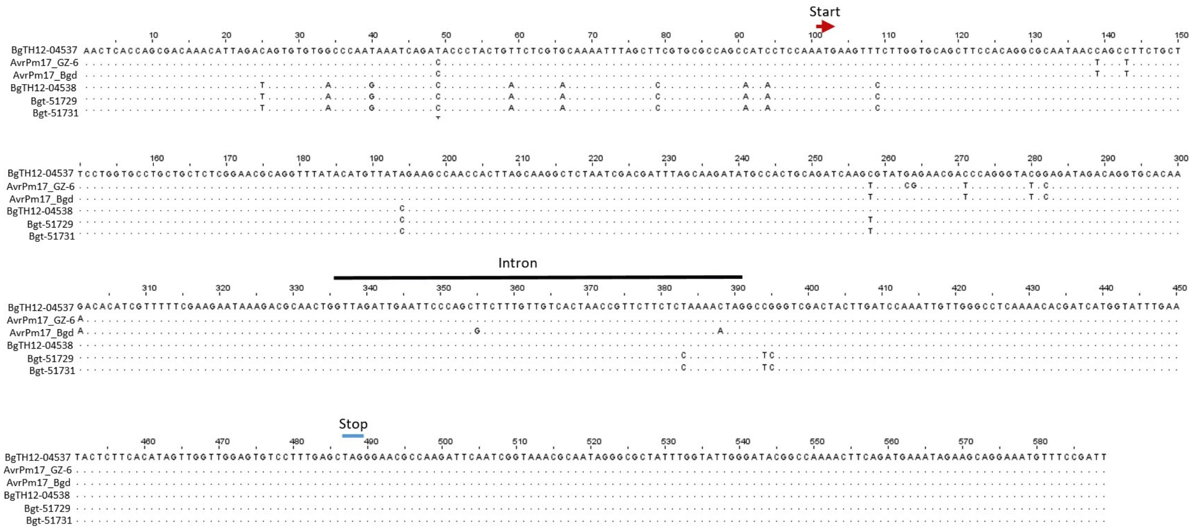

Fig. S14*.* Alignment of nucleotide sequences of *AvrPm17* in different *B. graminis* isolates. Alignment encompasses the coding sequences and 100bp up- and downstream of the start and stop codon respectively. Position of start and stop codons and the intron are indicated

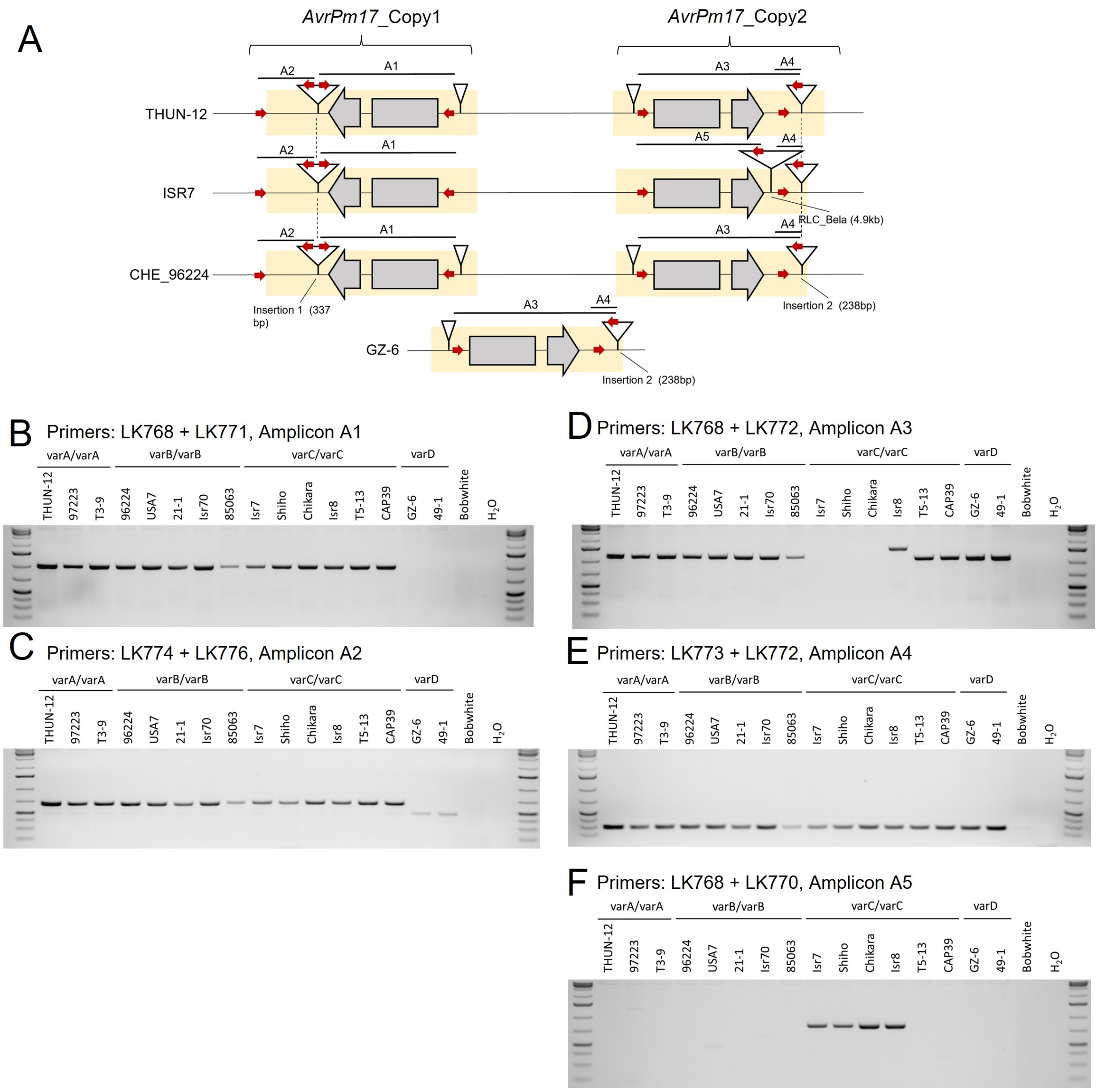

Fig. S15 PCR amplification of specific *AvrPm17* gene copies in a set of 16 isolates representing the four most frequent *AvrPm17* haplotypes present in the worldwide *B.g. tritici* population. A) Schematic representation of the *AvrPm17* locus in the high-quality genomes (PacBio) of THUN-12, ISR7 and Bgt_96224 as well as in the de-novo assembly (Illumina) of isolate GZ-6. The yellow box indicates the duplicated segment. Grey arrows indicate the *AvrPm17* gene copies. Red arrows indicate positions of primers used. For each primer pair, the expected amplicon (denotated A1-A5) is indicated by a black line. (B-E) Gel electrophoresis pictures showing amplification of amplicons A1-A5. All PCR products were Sanger sequenced to verify the *AvrPm17* coding sequence and the exact position of copy-specific insertions to be consistent with the respective reference assemblies shown in panel A. B) Amplification of copy 1 of *AvrPm17* using a primer (LK771) located in the specific insertion 1 of AvrPm17_Copy1. C) Amplification of the downstream region of copy 1 using a primer (LK774) located in the specific insertion 1 of *AvrPm17*_Copy1. D) Amplification of copy 2 of *AvrPm17* using a primer (LK772) located in the specific insertion 2 for *AvrPm17*_Copy2. There is no amplification in isolates carrying a 4.9kb insertion of a Copia retrotransposon RLC_Bela. Isolate Isr8 contains a truncated version of the RLC_Bela element. E) Amplification of the downstream region of copy 2 using a primer (LK772) located in the specific insertion 2 of

*AvrPm17*_Copy2. F) Amplification of copy 2 of *AvrPm17* in isolates carrying the Copia retrotransposon RLC_Bela, by using a primer (LK770) binding within the RLC_Bela element.

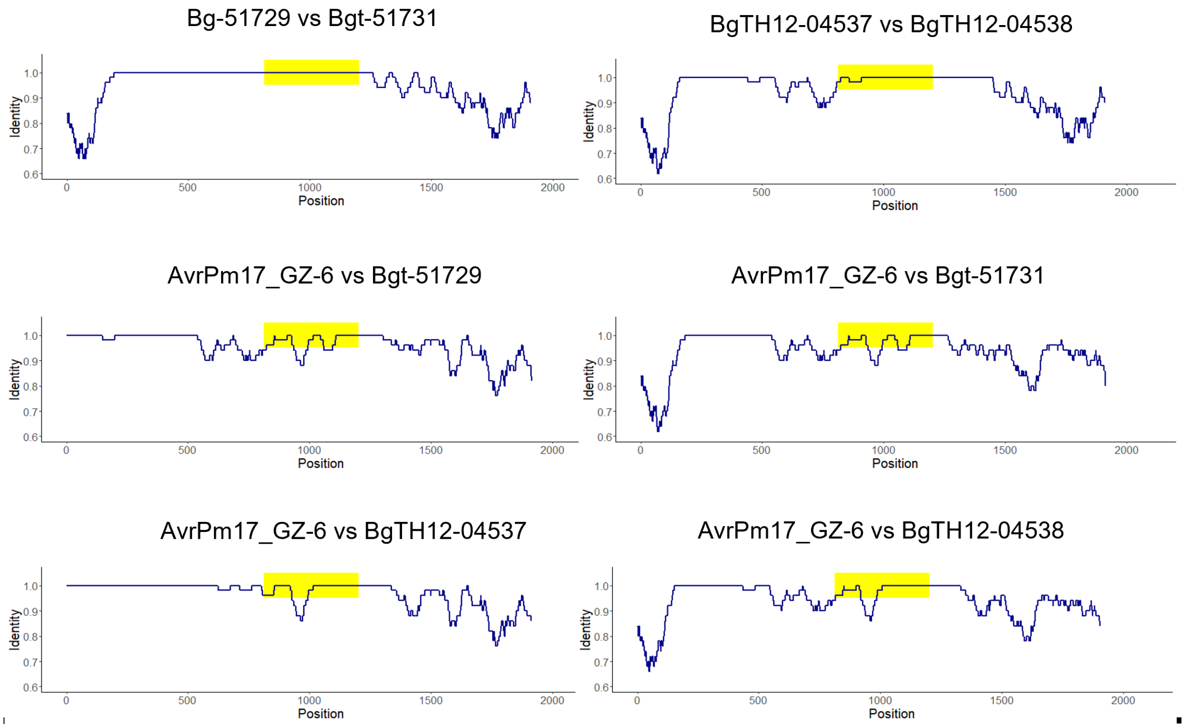

Fig. S16 Visual representation of sequence alignments of the *AvrPm17* gene duplication in different isolates. The x-axis shows the alignment position, while the y-axis shows the sequence identity calculated in 50bp sliding window. To facilitate alignment, the insertions/deletions in the sequences (see Fig. S15, Fig. S18) were removed. The position of the *AvrPm17* gene is highlighted in yellow. Note that in the alignment of *Bgt-51729* and *Bgt-51731* (first panel), the region containing the gene shows 100% identity while sequence identity decreases toward the end of the duplicated regions. The 200bp flanking region show 79% up and 84% downstream respectively. This suggests recurring gene conversions that keep the two copies of the gene identical.

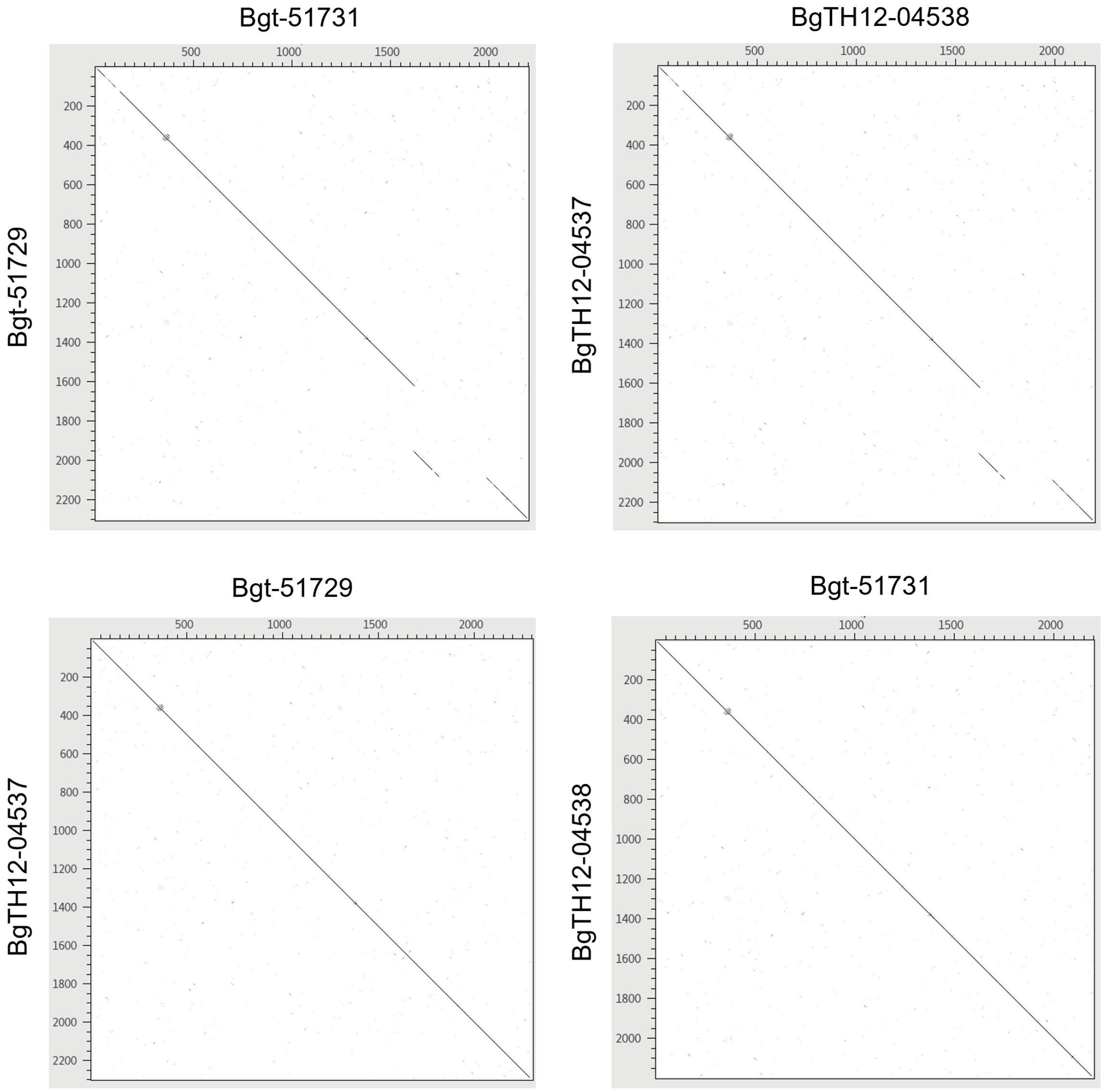

Fig. S17. Pairwise dotplots of the duplicated *AvrPm17* genes and flanking regions in the high-quality genomes of Bgt_96224 and THUN-12. The duplicated region spans 813 upstream of the start codons of the four genes and 1,079 and 998 downstream of the stop codon of *Bgt-51729/BgTH12-04537* and *Bgt-51731/BgTH12-04538*, respectively. The duplicated regions spanning 2,300bp for *Bgt-51729/BgTH12-04537* and 2200bp for *Bgt-51731/BgTH12-04538* were aligned using dotter.

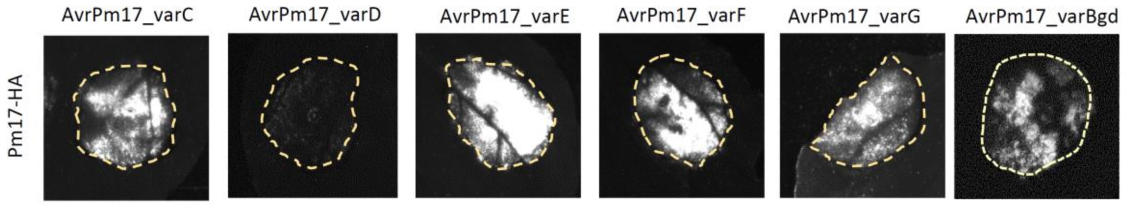

Fig. S18. Co-expression of *AvrPm17* haplovariants with *Pm17*-HA in *Nicotiana benthamiana*. Infiltrations were performed at infiltration ratio R:AVR of 1:4 and HR development was imaged using the Fusion Imager FX system after 5dpi. Effectors were considered as recognized if they induced an HR response under these standard conditions.

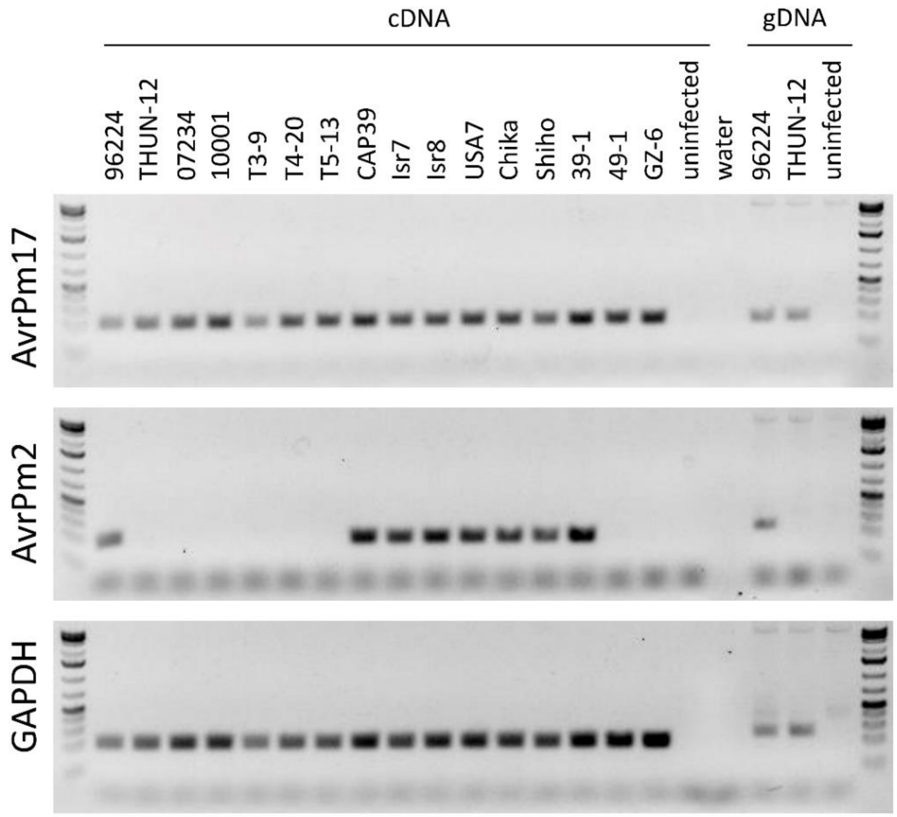

Fig. S19 *Pm17* is expressed in all tested isolates at 2dpi. Expression of the *AvrPm17* genes was tested using RT-PCR on cDNA originating from infected leaf segments of the susceptible cultivar Chinese Spring. Uninfected Chinese Spring was used as a control. RT-PCR amplification of the fungal housekeeping gene GAPDH and *AvrPm2*, which displays a well-characterized presence/absence polymorphism (13) are shown for comparison. PCR amplification of *AvrPm17, AvrPm2* and fungal *GAPDH* from genomic DNA of the two reference isolates Bgt_96224 and THUN-12 or uninfected Chinese Spring are shown for comparison. Amplicon size is slightly bigger on gDNA due to amplification over a small intron for all three tested genes.

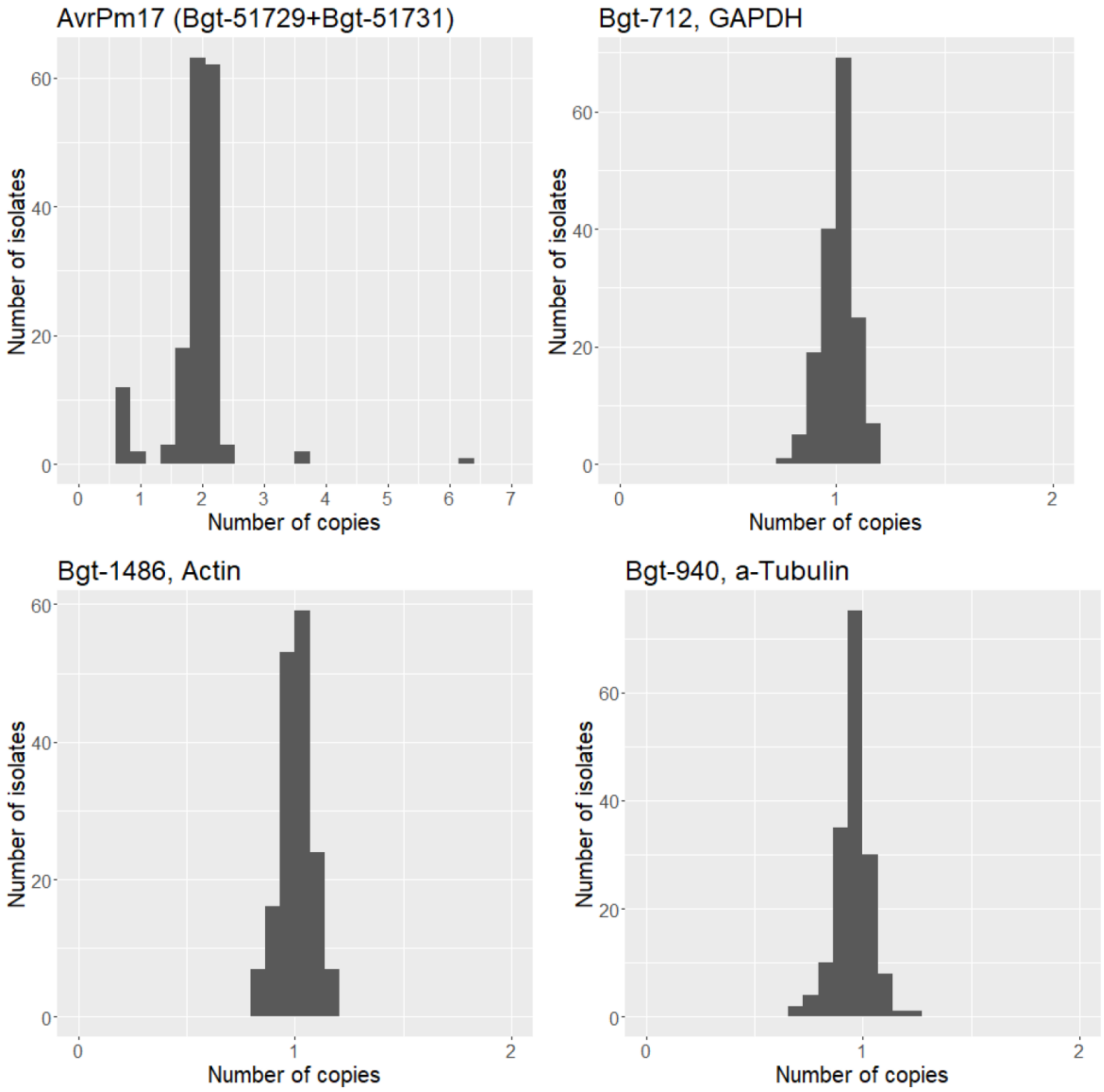

Fig. S20. The majority of *B.g. tritici*, *B.g. triticale* and *B.g. dicocci* isolates encode for two copies of *AvrPm17*. Copy number variation was estimated using genomic coverage of resequencing data normalized by the genomic coverage of all genes. As control, the normalized genomic coverage of three housekeeping genes (*GAPDH*, *Actin* and *α-Tubulin*) are shown.

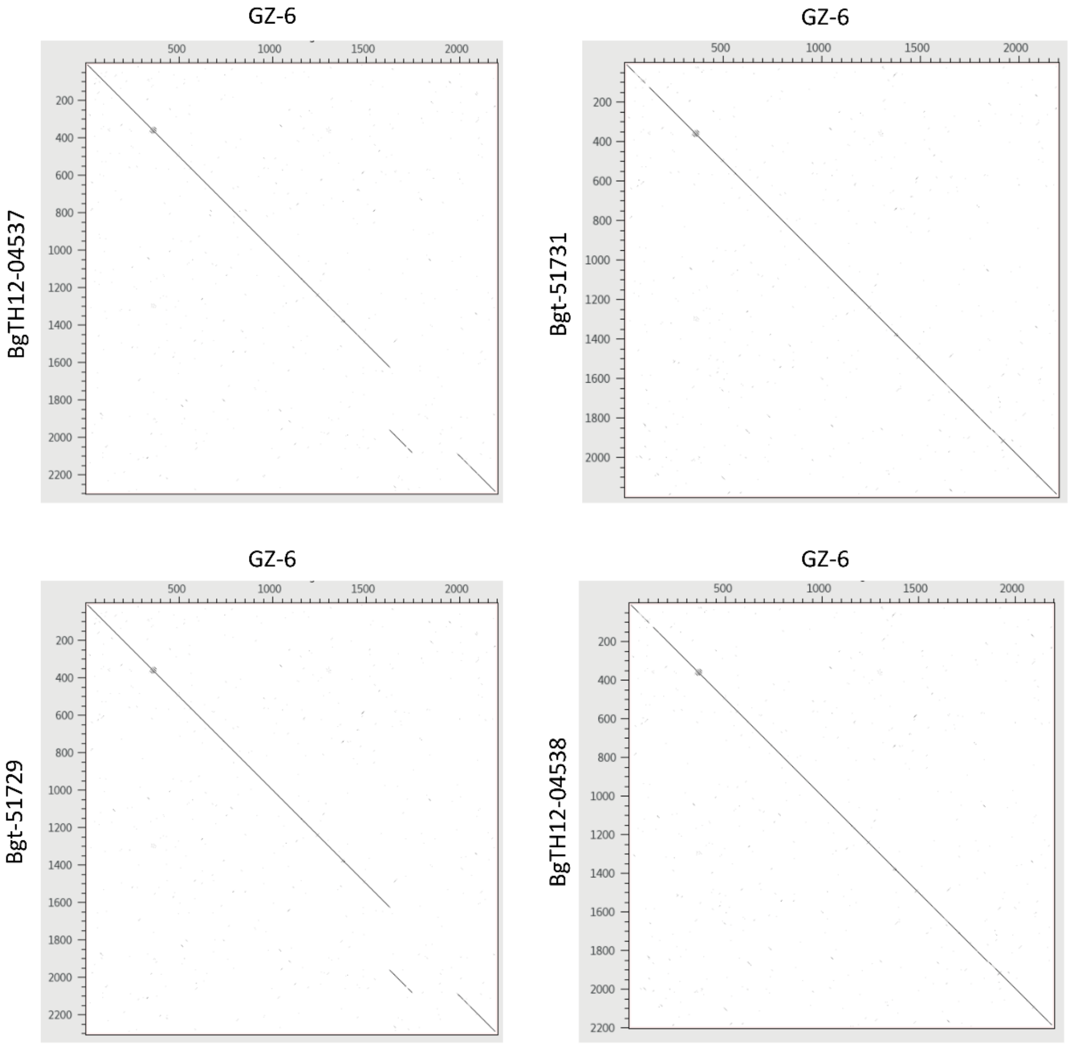

Fig. S21. Pairwise dotplots of the duplicated *AvrPm17* genes and flanking region in the high-quality genome of Bgt_96224 and THUN-12 with the *AvrPm17* gene copy of isolate GZ-6**.** Isolate GZ-6 contains only one *AvrPm17* copy. The upstream flanking region of this gene is more similar to *BgTH12-04537/Bgt-51729* as shown in the left panels, whereas the downstream region of the gene in GZ-6 is more similar to *BgTH12-04538/Bgt-51731* as shown in the right panels. This indicates that the *AvrPm17* gene copy is a recombinant between the two duplicated gene copies.

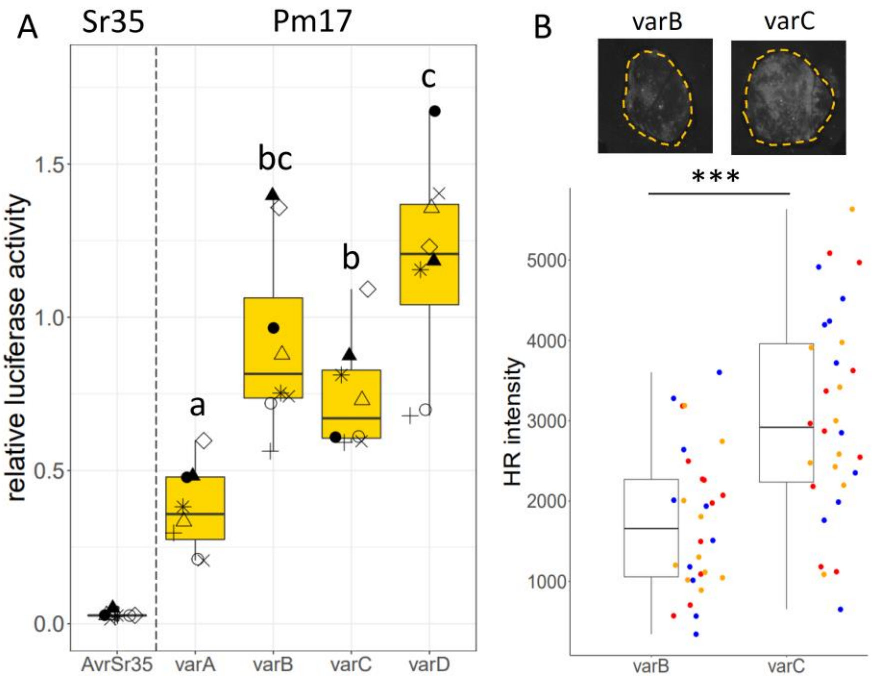

Fig. S22 Cell death response upon co-expression of different *AvrPm17* variants with *Pm17* in wheat protoplasts and *Nicotiana benthamiana*. (A) Co-transfection of *Pm17* with different *AvrPm17* variants in wheat protoplasts (cv. “Chinese Spring”). All samples where co-transfected with a *pUBQ:luciferase* construct and relative luciferase activity after 16h was used to estimate the amplitude of cell death (i.e reduction of protoplast viability). Luciferase signal was normalized to *Pm17* co-transfected with empty vector (pIPKb002). Results from eight independent co-transfections per R/Avr combination are depicted and individual datapoints indicated with different symbols. Statistical difference between *AvrPm17* variants was assessed using ANOVA followed by TukeyHSD post-hoc test. Different letters above the boxplot indicate statistical significance at $p<0.05$. (B) Recognition strength of AVRPM17_varB compared to AVRPM17_varC in *Nicotiana benthamiana*. Infiltrations were performed at R: Avr ratios of 1:1 and significance was assessed using a paired Wilcoxon rank sum test. Significance level is indicated above the boxplots as follows: *** $p<0.001$. Y-axis represent quantitative measurement of HR based on the Fusion FX imager system. Individual datapoints are color-coded based on three independent experiments with at least n=8 leaves per experiment.

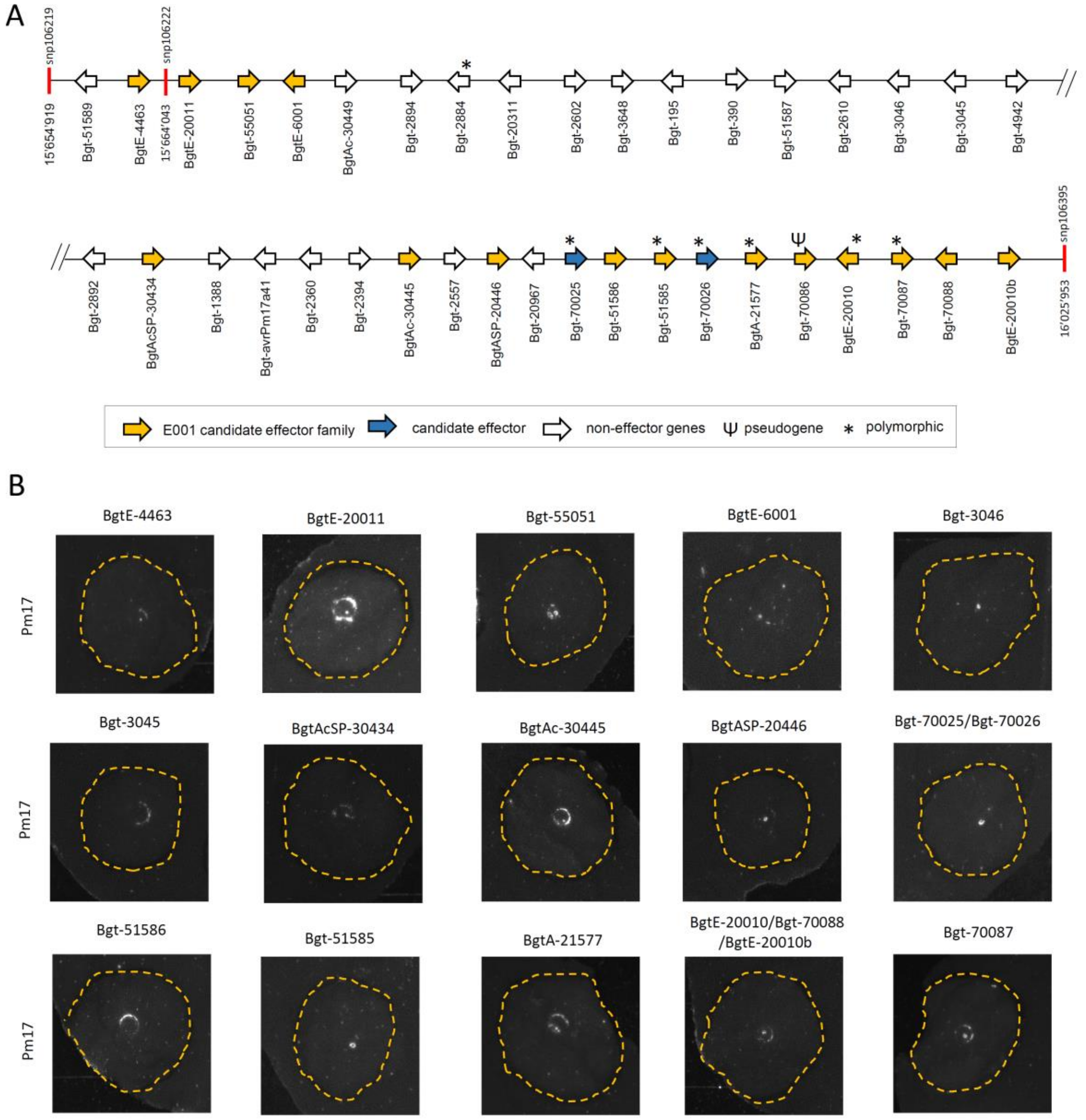

Figure S23. QTL on chromosome 9 contains numerous effector candidates but does not encode a *Pm17* avirulence component. (A) Schematic representation of the physical interval underlying the genetic confidence interval (LOD=1.5) on chromosome 9 on the assembly of the avirulent isolate Bgt_96224. Genes and orientation are indicated; gene size is not drawn to scale. Polymorphic genes between parental isolates Bgt_96224 and THUN-12 are indicated by an asterisk. Bgt-70025 and Bgt-70026 represent genes that contain a signal peptide and show no homology outside the genus *Blumeria* and therefore likely represent candidate effectors. (B) *N. benthamiana* co-expression assays with Pm17-HA of the 16 effector genes and two putatively secreted non-effector genes found within the confidence interval depicted in panel (A). Infiltrations were performed at a ratio of Pm17: candidate gene of 1:4 and images were taken 5 days after infiltration using the FX Fusion Imager system.

Table S1. Results of the QTL mapping

Line	Chr	Marker[a]	cM[b]	LOD[c]	Pos[d]	1.5LOD[e]	Interval Bgt_96224[f]	Interval Bgt_96224 with parametric test[g]	Interval THUN-12[h]
BW Pm17#34	Bgt_chr-01	snp4585	164.8	9.2	4390396	snp4525 - snp4614	4'338'270 -4'452'554	4’359’577 -4’447’240	4'085'291 -4'147'189
BW Pm17#181	Bgt_chr-01	snp4552 - snp4584	164.8	7.0	4361087 -4390173	snp4394 - snp4622	4'166'104 -4'458'811	4’124’946 -4’503’787	3'921'779 -4'159'203

[a]Best associated marker in the single interval QTL analysis. It is possible that several markers are equally significant
[b]cM position at the best associated marker
[c]Logarithm of the odds. Significance LOD threshold was calculated by 1000 permutations
[d]Position of the best associated SNP in the Bgt_genome_v3_16 assembly
[e]Markers delimitating the genetic confidence interval (1.5LOD interval)
[f]Physical interval underlying the genetic confidence interval in the *B.g. tritici* assembly Bgt_genome_v3_16
[g]Physical interval underlying the LOD 1.5 genetic confidence interval in the *B.g. tritici* assembly Bgt_genome_v3_16 using a parametric test performed with the scanone(method=”normal”) command
[h]Physical interval underlying the genetic confidence interval in the *B.g. triticale* assembly THUN12_genome_v1

Table S2. Gene orientation of duplicated candidate effector genes in Bgt_96224 that encode identical proteins

Genename	Orientation
Bgt-20382,Bgt-55090	not on same chromosome
Bgt-51729,Bgt-51731	inverted
Bgt-55088,BgtE-5867	tandem
Bgt-55087,BgtE-5861	tandem
Bgt-50289,Bgt-50587,Bgt-50590	not on same chromosome
Bgt-51188,Bgt-55139,Bgt-55140	Bgt-55139 and Bgt-55140 in tandem, Bgt-51188 not adjacent
Bgt-51503,Bgt-51504	tandem
Bgt-50967,Bgt-51338	inverted
Bgt-55072,BgtE-10014)	inverted d
Bgt-55073,Bgt-55074,Bgt-55075	tandem
Bgt-51063,Bgt-51064	tail-to-tail, not adjacent
Bgt-55024,Bgt-55025,BgtE-20071	BgtE-20071 and Bgt-55024 in tandem, Bgt-55025 not adjacent
Bgt-BCG-2,Bgt-55094	not on same chromosome
Bgt-55109,Bgt-55048	inverted
Bgt-55041,Bgt-55119	inverted

Table S3. Investigation of *Pm17* in publicly available rye genomic resources

Genotype	Resource	Publication	Presence *Pm17*
Lo7	Assembly	Rabanus-Wallace et al., 2021	no
Weining	Assembly	Li et al., 2021	no
Lo90	WGS	Bauer et al., 2017	no
Lo115	WGS	Bauer et al., 2017	no
Lo117	WGS	Bauer et al., 2017	no
Lo176	WGS	Bauer et al., 2017	no
Lo191	WGS	Bauer et al., 2017	no
Lo282	WGS	Bauer et al., 2017	yes
Lo298	WGS	Bauer et al., 2017	yes
Lo310	WGS	Bauer et al., 2017	no
Lo348	WGS	Bauer et al., 2017	yes
Lo351	WGS	Bauer et al., 2017	yes
Svavi	WGS	Bauer et al., 2017	no

Table S4. Nucleotide identity in the 200bp distal region of the gene duplication in different isolates. Upper panel represents value of the upstream region of the duplication, lower panel represent the region downstream of the genes.

	Bgt-51729	BgTH12-04537	AvrPm17_GZ-6	BgTH12-04538	Bgt-51731
Bgt-51729	100	99.5	99.5	78	79
BgTH12-04537	99.5	100	100	78.5	79.5
AvrPm17_GZ-6	99.5	100	100	78.5	79.5
BgTH12-04538	78	78.5	78.5	100	99
Bgt-51731	79	79.5	79.5	99	100

	Bgt-51729	BgTH12-04537	Bgt-51731	BgTH12-04538	AvrPm17_GZ-6
Bgt-51729	100	99.49	84	84	86.5
BgTH12-04537	99.49	100	83.33	83.33	85.86
Bgt-51731	84	83.33	100	100	90
BgTH12-04538	84	83.33	100	100	90
AvrPm17_GZ-6	86.5	85.86	90	90	100

Table S5. Phenotypes of natural *B.g. tritici* and *B.g. triticale* isolates on *Pm17* transgenic lines and Matador rye.

f.sp.	Haplotype[a]	Sis#34	Pm17#34[b]	Sis#181	Pm17#181[b]	Matador	Isolate
B.g. tritici	varA/varA	1	0.00	1	0.00	0.00	97223
B.g. triticale	varA/varA	n.t.	n.t.	1	0.00	weak[c]	T3-9
B.g. tritici	varA/varC	1	0.46	1	0.17	n.t.	10001
B.g. tritici	varB/varB	1	0.90	1	0.92	0.00	21-1
B.g. tritici	varB/varB	1	0.71	1	0.54	0.00[c]	Isr70
B.g. tritici	varB/varB	1	0.33	1	0.25	0.00	85063
B.g. tritici	varB/varB	1	0.59	1	0.45	0.00	Usa7
B.g. tritici	varC/varC	0.97	0.20	0.98	0.06	0.00[c]	Isr7
B.g. triticale	varC/varC	1	0.83	1	0.29	00.0	T5-13
B.g. tritici	varC/varC	1	0.83	1	0.42	0.00[c]	Isr8
B.g. tritici	varC/varC	1	0.51	1	0.15	0.00	Shiho
B.g. triticale	varC/varC	1	0.33	1	0.00	0.00[c]	CAP-39
B.g. tritici	varC/varC	n.t.	0.38	n.t.	0.44	0.00	Chikara
B.g. tritici	varD	1	1	1	0.98	0.00	GZ-6
B.g. tritici	varD	1	0.97	1	0.98	0.00	49-1

[a] AVRPM17 haplotype combination encoded by the isolate

[b] Leaf coverage of individual leaf segments was scored according to the following scale: avirulent = 0, avirulent/intermediate 0.25, intermediate 0.5, intermediate/virulent =0.75, virulent = 1. For each isolate, the average of at least 3 individual leaf segments is indicated.

[C] phenotypes published in Menardo et al., 2016

Table S6. Results of the QTL mapping based on 117 progeny of the cross Bgt_96224 X THUN12

Line	Chr	Marker[a]	cM[b]	LOD[c]	Pos[d]	1.5LOD[e]	Interval Bgt_96224[f]	Interval Bgt_96224 with parametric test[g]	Interval THUN-12[h]	Origin[i]
Amigo	Bgt_chr-09	c9.loc340	340.0	15.97	15’664’043	snp106219	15’654’919	15’654’919	15’628’219	*B.g. tritici*
						snp106395	16’025’953	15’914’562	16’002’212	
Amigo	Bgt_chr-01	snp4594 - snp4610	165.8	4.24	4’357’972	snp4389	4’157’551	4’157’551	3’913228	*B.g. tritici*
						snp4685	4’530’020	4’530’020	4’233’204	

[a]Best associated Marker in the single interval QTL analysis

[b]cM position of the best associated marker

[c]Logarithm of the odds, significance LOD threshold was calculated by 1000 permutations

[d]Position of best associated SNP in the *B.g. tritici* assembly Bgt_genome_v3_16

[e]Markers delimitating the genetic confidence interval (1.5LOD interval)

[f]Physical interval underlying the genetic confidence interval in the *B.g. tritici* assembly Bgt_genome_v3_16

[g] Physical interval underlying the LOD 1.5 genetic confidence interval in the *B.g. tritici* assembly Bgt_genome_v3_16 using a parametric test performed with the scanone(method=”normal”) command

[h]Physical interval underlying the genetic confidence interval in the *B.g. tritici* assembly THUN-12_genome_v1_1

[i]Origin of the interval in *B.g. triticale* THUN-12 based on Müller et al., 2021

Table S7. ***Summary of genes in genetic confidence interval on chromosome 9***

Name	SP[a]	Polymorphic[b]	Gene description[c]	Effector family[d]	logFC[e]	Expression 96224[f]	Expression THUN-12[g]	Tested[h]
Bgt-51589	-	-	-	-	-	0.0	0.0	
BgtE-4463	-	-	Candidate effector	E001	0.00	19.4	19.4	yes
BgtE-20011	-	-	Candidate effector	E001	-0.09	47.3	46.5	yes
Bgt-55051	yes	-	Candidate effector	E001	-0.49	37.0	27.1	yes
BgtE-6001	yes	-	Candidate effector	-	-0.36	52.7	41.7	yes
BgtAc-30449	-	-	-	-	-0.41	51.4	40.6	
Bgt-2894	-	-	Putative ntf2-like domain containing protein	-	0.08	124.6	140.7	
Bgt-2884	-	1	DUF962-domain containing protein	-	0.09	141.0	155.0	
Bgt-20311	-	-	RIX_Bgt_Tara	-	-0.62	55.7	38.0	
Bgt-2602	-	-	GTPase-activating protein	-	0.36	16.6	23.3	
Bgt-3648	-	-	-	-	0.63	11.6	18.4	
Bgt-195	-	-	O-mannosyltransferase	-	-0.04	103.2	105.0	
Bgt-390	-	-	Dipeptidyl-aminopeptidase B	-	0.11	42.1	48.5	
Bgt-51587	-	-	-	-	-	0.0	0.0	
Bgt-2610	-	-	Ribokinase-like protein	-	0.13	122.3	141.4	
Bgt-3046	yes	-	putative upf0480 protein	-	-0.46	110.9	83.3	yes
Bgt-3045	yes	-	MARVEL-like domain protein	-	-0.88	58.8	33.3	yes
Bgt-4942	-	-	O-acetylhomoserine (thiol)-lyase	-	-0.16	41.1	39.1	
Bgt-2892	-	-	RNA polymerase II subunit B16	-	-0.51	209.1	157.1	
BgtAcSP-30434	yes	-	Candidate effector	E001	-	2.2	0.7	yes
Bgt-1388	-	-	Ergosterol biosynthesis	-	-1.18	299.2	139.3	
Bgt-avrPm17a41	-	-	Putative RNA-binding protein fus tls protein	-	0.02	99.8	107.0	
Bgt-2390	-	-	Rab2/secretion related GTPase	-	-0.12	37.3	36.0	
Bgt-2394	-	-	Choline transporter-like protein	-	0.29	42.3	54.0	
BgtAc-30445	-	-	Candidate effector	-	-0.14	12.6	11.7	yes
Bgt-2557	-	-	Transcription factor	-	-0.03	31.4	33.8	
BgtASP-20446	yes	-	Candidate effector	E001	-0.40	48.0	38.2	yes
Bgt-20967	-	-	No open reading frame	-	-	21.8	18.4	
Bgt-70025	yes	2	Candidate effector	NC	0.60	39.7	67.0	yes

Bgt-51586	yes	-	Candidate effector	E001	0.31	18.9	24.7	yes
Bgt-51585	yes	2	Candidate effector	E001	-0.11	56.6	54.9	yes
Bgt-70026	yes	1	Candidate effector	NC	-0.86	21.7	12.3	yes
BgtA-21577	-	4,0[i]	Candidate effector	E001	0.63	22.9	36.3	yes
Bgt-70086	-	-	Candidate effector, premature stop codon	-	-2.47	66.6	12.7	
BgtE-20010	-	1	Candidate effector	E001	-1.04	515.9	263.1	yes
Bgt-70087	-	1,7[i]	Candidate effector	E001	-0.68	224.5	147.9	yes
Bgt-70088	-	-	Candidate effector	E001	-1.04	515.9	263.1	yes
BgtE-20010b	-	-	Candidate effector	E001	-1.04	515.9	263.1	yes

[a]Signal peptide prediction according to SignalP5.0
[b]Number of amino acid polymorphisms between the parental isolates 96224 and THUN-12
[c]Classification of gene into candidate effector (see (2)). For non-effector genes, the protein was blasted against the NCBI database, putative function is indicated
[d]Effector gene family definition according to (2). NC designates genes that were newly annotated compared to the annotation presented in (2) but like effectors contain a signal peptide and show no homology outside the genus.
[e]Differential gene expression analysis comparing expression levels in *B.g. tritici* 96224 on the susceptible wheat cultivar ‘Chinese Spring’ at 2dpi with *B.g. triticale* THUN-12 on the susceptible triticale cultivar ‘Timbo’. Expression differences are indicated as logFC. logFC >1.5 was considered significant. Genes with missing logFC values are not expressed.
[f]Average gene expression of three biological replicates in isolate 96224 at 2dpi on the susceptible wheat cultivar ‘Chinese Spring’. Expression values are indicated as rpkm (Reads Per Kilobase of transcript, per Million mapped reads).
[g]Average gene expression of three biological replicates in isolate THUN-12 at 2dpi on the susceptible triticale cultivar ‘Timbo’. Expression values are indicated as rpkm (Reads Per Kilobase of transcript, per Million mapped reads).
[h]Tested in co-expression assays with *Pm17*-HA in *Nicotiana benthamiana.*
[i]Gene is present in two copies in isolate THUN-12.

Dataset S1 (separate file). Sequence of effector genes used for expression in *N. benthamiana*

Dataset S2 (separate file). List of primers used in this study

Dataset S3 (separate file). Information about *B.graminis* isolates used in this study

SI References

1. S. Fouche, C. Plissonneau, D. Croll, The birth and death of effectors in rapidly evolving filamentous pathogen genomes. *Current Opinion in Microbiology* **46**, 34-42 (2018).
2. M. C. Müller *et al.*, A chromosome-scale genome assembly reveals a highly dynamic effector repertoire of wheat powdery mildew. *New Phytologist* **221**, 2176-2189 (2019).
3. J.-M. Chen, D. N. Cooper, N. Chuzhanova, C. Ferec, G. P. Patrinos, Gene conversion: mechanisms, evolution and human disease. *Nature Reviews Genetics* **8**, 762-775 (2007).
4. H. Liu *et al.*, Tetrad analysis in plants and fungi finds large differences in gene conversion rates but no GC bias. *Nature Ecology & Evolution* **2**, 164-173 (2018).
5. S. Dong *et al.*, The *Phytophthora sojae* avirulence locus Avr3c encodes a multi-copy RXLR effector with sequence polymorphisms among pathogen strains. *Plos One* **4**, e5556 (2009).
6. M. Möller, E. H. Stukenbrock, Evolution and genome architecture in fungal plant pathogens. *Nature Reviews Microbiology* **15**, 756 – 771 (2017).
7. S. Bourras *et al.*, The AvrPm3-Pm3 effector-NLR interactions control both race-specific resistance and host-specificity of cereal mildews on wheat. *Nature Communications* **10**, 2292 (2019).
8. S. P. Singh *et al.*, Evolutionary divergence of the rye Pm17 and Pm8 resistance genes reveals ancient diversity. *Plant Molecular Biology* **98**, 249-260 (2018).
9. J. R. Lowry, D. J. Sammons, P. S. Baenziger, J. G. Moseman, Identification and characterization of the gene conditioning powdery mildew resistance in Amigo wheat. *Crop Science* **24**, 129-132 (1984).
10. E. L. Olson *et al.*, Genotyping of US wheat germplasm for presence of stem rust resistance genes *Sr24*, *Sr36* and *Sr1RS*(Amigo). *Crop Science* **50**, 668-675 (2010).
11. RStudio-Team (2018) RStudio: integrated development environment for R. (http://www.rstudio.com/).
12. M. C. Müller, L. Kunz, J. Graf, S. Schudel, B. Keller, Host adaptation through hybridization: genome analysis of triticale powdery mildew reveals unique combination of lineage-specific effectors. *Molecular Plant-Microbe Interactions* **34**, 1350-1357 (2021).
13. C. R. Praz *et al.*, AvrPm2 encodes an RNase-like avirulence effector which is conserved in the two different specialized forms of wheat and rye powdery mildew fungus. *New Phytologist* **213**, 1301-1314 (2017).

14. F. Menardo *et al.*, Hybridization of powdery mildew strains gives rise to pathogens on novel agricultural crop species. *Nature Genetics* **48**, 201-205 (2016).
15. A. Dobin *et al.*, STAR: ultrafast universal RNA-seq aligner. *Bioinformatics* **29**, 15-21 (2013).
16. C. R. Praz *et al.*, Non-parent of Origin Expression of Numerous Effector Genes Indicates a Role of Gene Regulation in Host Adaption of the Hybrid Triticale Powdery Mildew Pathogen. *Frontiers in Plant Science* **9**, 49 (2018).
17. J. T. Robinson *et al.*, Integrative genomics viewer. *Nature Biotechnology* **29**, 24-26 (2011).
18. J. J. A. Armenteros *et al.*, SignalP 5.0 improves signal peptide predictions using deep neural networks. *Nature Biotechnology* **37**, 420-+ (2019).
19. R. Patro, G. Duggal, M. I. Love, R. A. Irizarry, C. Kingsford, Salmon provides fast and bias-aware quantification of transcript expression. *Nature Methods* **14**, 417-+ (2017).
20. M. D. Robinson, D. J. McCarthy, G. K. Smyth, edgeR: a Bioconductor package for differential expression analysis of digital gene expression data. *Bioinformatics* **26**, 139-140 (2010).
21. A. Himmelbach, U. Zierold, G. Hensel, e. al. (2007) A set of modular binary vectors for transformation of cereals. (*Plant Physiol*.), pp 1192-1200.
22. D. Weigel, J. Glazebrook, Transformation of agrobacterium using the freeze-thaw method. *CSH protocols* **2006** (2006).
23. J. Schindelin *et al.*, Fiji: an open-source platform for biological-image analysis. *Nature Methods* **9**, 676-682 (2012).
24. S. G. Milner *et al.*, Genebank genomics highlights the diversity of a global barley collection. *Nature Genetics* **51**, 319-+ (2019).
25. S. G. Milner *et al.*, Genebank genomics highlights the diversity of a global barley collection. *Nature Genetics* **51**, 319–326 (2019).
26. E. Garrison, G. Marth (2012) Haplotype-based variant detection from short-read sequencing. (arXiv:), p 1207.3907v1202.
27. P. Danecek *et al.*, The variant call format and VCFtools. *Bioinformatics* **27**, 2156-2158 (2011).
28. L. Zimmermann *et al.*, A completely reimplemented MPI bioinformatics toolkit with a new HHpred server at its core. *Journal of Molecular Biology* **430**, 2237-2243 (2018).
29. L. J. McGuffin *et al.*, IntFOLD: an integrated web resource for high performance protein structure and function prediction. *Nucleic Acids Research* **47**, W408-W413 (2019).
30. C. Camacho *et al.*, BLAST plus : architecture and applications. *Bmc Bioinformatics* **10**, 421 (2009).
31. R. C. Edgar, MUSCLE: a multiple sequence alignment method with reduced time and space complexity. *Bmc Bioinformatics* **5**, 1-19 (2004).
32. M. N. Price, P. S. Dehal, A. P. Arkin, FastTree 2-Approximately Maximum-Likelihood trees for large alignments. *Plos One* **5**, e9490 (2010).
33. M. N. Price, P. S. Dehal, A. P. Arkin, FastTree 2-Approximately Maximum-Likelihood Trees for Large Alignments. *Plos One* **5** (2010).

34. D. M. Emms, S. Kelly, OrthoFinder: phylogenetic orthology inference for comparative genomics. *Genome Biology* **20** (2019).
35. L. Frantzeskakis *et al.*, Signatures of host specialization and a recent transposable element burst in the dynamic one-speed genome of the fungal barley powdery mildew pathogen. *Bmc Genomics* **19** (2018).
36. H. Li *et al.*, The Sequence Alignment/Map format and SAMtools. *Bioinformatics* **25**, 2078-2079 (2009).
37. S. Bourras *et al.*, Multiple avirulence loci and allele-specific effector recognition control the Pm3 race-specific resistance of wheat to powdery mildew. *Plant Cell* **27**, 2991-3012 (2015).
38. B. Langmead, S. L. Salzberg, Fast gapped-read alignment with Bowtie 2. *Nature Methods* **9**, 357- 359 (2012).
39. A. M. Bolger, M. Lohse, B. Usadel, Trimmomatic: a flexible trimmer for Illumina sequence data. *Bioinformatics* **30**, 2114-2120 (2014).
40. D. M. Emms, S. Kelly, OrthoFinder: phylogenetic orthology inference for comparative genomics. *Genome Biology* **20**, 238 (2019).
41. L. Frantzeskakis *et al.*, Signatures of host specialization and a recent transposable element burst in the dynamic one-speed genome of the fungal barley powdery mildew pathogen. *Bmc Genomics* **19**, 381 (2018).
42. E. L. L. Sonnhammer, R. Durbin, A dot-matrix program with dynamic threshold control suited for genomic DNA and protein sequence analysis. *Gene-Combis* **167**, 1-10 (1995).
43. A. Bankevich *et al.*, SPAdes: A new genome assembly algorithm and its applications to single-cell sequencing. *Journal of Computational Biology* **19**, 455-477 (2012).
44. M. T. Rabanus-Wallace *et al.*, Chromosome-scale genome assembly provides insights into rye biology, evolution and agronomic potential. *Nature genetics* **53**, 564–573 (2021).
45. G. Li *et al.*, A high-quality genome assembly highlights rye genomic characteristics and agronomically important genes. *Nature genetics* **53**, 574–584 (2021).
46. E. Bauer *et al.*, Towards a whole-genome sequence for rye (Secale cereale L.). *Plant Journal* **89**, 853-869 (2017).
47. H. Li, R. Durbin, Fast and accurate short read alignment with Burrows-Wheeler transform. *Bioinformatics* **25**, 1754-1760 (2009).
48. A. Förderer *et al.*, A wheat resistosome defines common principles of immune receptor channels. *bioRxiv*, 2022.2003.2023.485489 (2022).
49. I. M. L. Saur, S. Bauer, X. Lu, P. Schulze-Lefert, A cell death assay in barley and wheat protoplasts for identification and validation of matching pathogen AVR effector and plant NLR immune receptors. *Plant Methods* **15** (2019).
50. H. Kobayashi *et al.*, X-Ray crystallographic structure of RNase Po1 that exhibits antitumor activity. *Biological & Pharmaceutical Bulletin* **37**, 968-978 (2014).
51. H. G. Pennington *et al.*, The fungal ribonuclease-like effector protein CSEP0064/BEC1054 represses plant immunity and interferes with degradation of host ribosomal RNA. *Plos Pathogens* **15** (2019).
